# Catalogue, distribution, taxonomic notes, and conservation of the Western Palearctic endemic hunchback beetles (Tenebrionidae, *Misolampus*)

**DOI:** 10.3897/zookeys.963.53500

**Published:** 2020-08-24

**Authors:** Natalia Rosas-Ramos, Paloma Mas-Peinado, Diego Gil-Tapetado, Ernesto Recuero, José L. Ruiz, Mario García-París

**Affiliations:** 1 Departamento de Biología Animal (Área de Zoología), Facultad de Biología (Edificio de Farmacia, planta 5), Universidad de Salamanca, Campus Miguel de Unamuno s/n, 37007 Salamanca, Spain Museo Nacional de Ciencias Naturales Madrid Spain; 2 Departamento de Biodiversidad y Biología Evolutiva. Museo Nacional de Ciencias Naturales, MNCN-CSIC. c/ José Gutiérrez Abascal, 2. 28006, Madrid, Spain Universidad de Salamanca Salamanca Spain; 3 Centro de Investigación en Biodiversidad y Cambio Global CIBC-UAM, Facultad de Ciencias, Universidad Autónoma de Madrid, c/Darwin 2, 28049-Madrid, Spain Universidad Autónoma de Madrid Madrid Spain; 4 Departamento de Biología, Ecología y Evolución, Facultad de Ciencias Biológicas, Universidad Complutense de Madrid, c/ José Antonio Novais, 12, 28040-Madrid, Spain Universidad Complutense de Madrid Madrid Spain; 5 Instituto de Estudios Ceutíes. Paseo del Revellín, 30. 51001 Ceuta, Spain Instituto de Estudios Ceutíes Ceuta Spain

**Keywords:** Coleoptera, geographic range, morphological variability, new synonymies, population persistence, saproxylic, scientific collections, Stenochiinae

## Abstract

Hunchback darkling beetles of the Ibero-Maghrebian genus *Misolampus* Latreille, 1807 (Tenebrionidae, Stenochiinae) encompass six species: *M. gibbulus* (Herbst, 1799), *M. goudotii* Guérin-Méneville, 1834, *M. lusitanicus* Brême, 1842, *M. ramburii* Brême, 1842, *M. scabricollis* Graells, 1849, and *M. subglaber* Rosenhauer, 1856. Previously known distribution ranges of the species were delineated using many old records, the persistence of such populations being questionable under the current situation of global biodiversity loss. Additionally, the status of geographically isolated populations of the genus have been the subject of taxonomic controversy. An exhaustive bibliographical revision and field search was undertaken, and the *Misolampus* collection of the Museo Nacional de Ciencias Naturales (MNCN-CSIC) was revised. The aims are to (i) provide an updated geographic distribution range for the species of *Misolampus*; (ii) to determine the taxonomic status of controversial populations; (iii) to provide a catalogue for *Misolampus*; and (iv) to discuss the conservation status of these saproxylic beetles. As a result, a catalogue including synonymies and type localities, geographical records, diagnoses, and information on natural history for all species of *Misolampus* is presented. The results reveal that the distribution ranges of the species of *Misolampus* have not undergone a reduction in the last century, and indicate the presence of the genus in areas where it had never been recorded before. The morphological variability of *M. goudotii* drove the proposal of different taxa that are here formally synonymised as follows: *M. goudotii* Guérin-Méneville, 1834 = *M. erichsoni* Vauloger de Beaupré, 1900, **syn. nov.** = *M. peyerimhoffi* Antoine, 1926, **syn. nov.**

## Introduction

Species identification is an essential process for almost all biodiversity studies and can constitute a major constraint for conservation evaluation and legislation due to the inherent difficulty of identifying many of the groups, the long time needed for processing the samples, and the extensive taxonomic experience that this process requires (Gauld et al. 2000; Guerra-García et al. 2008; Wheeler 2013; de Oliveira et al. 2020; Saoud 2020). Meeting this goal for megadiverse groups such as insects is often arduous considering the vast number of species that must be identified and the limited number of taxonomists, which make correct identification a very time-consuming process (De Carvalho et al. 2005; Evenhuis 2007; Wheeler 2008; Moore 2011; Yang et al. 2015). It is therefore necessary to create easy-to-use identification tools, such as visually enhanced guides, to overcome the difficulties involved in the identification process (Kirchoff et al. 2011). Easy-to-use tools are also a key instrument for biodiversity study and conservation, since they can be used by both specialists and non-specialists and their implementation improves outcome, thereby facilitating decision-making for conservation actions (Norton et al. 2000; Yang et al. 2015; Rosas-Ramos et al. 2019).

Tenebrionidae is one of the most species-rich families of beetles, with approximately 20,000 species worldwide and many more taxa yet to be described (Bousquet et al. 2018). The large number of species, combined with the high morphological diversity that this family exhibits (Matthews et al. 2010), can hinder species identification of tenebrionids. Thus, providing easy-to-use, photographically illustrated identification tools can greatly facilitate data acquisition on this group of beetles (e.g., Matthews and Bouchard 2008; Pérez-Vera and Ávila 2012). The problem represented by the local absence of taxonomists and lack of adequate identification tools for non-specialists, is often reflected in a general lack of appropriate identifications, use of not-actualised names, or worst, inclusion of misidentified specimens in scientific collections or databases (Vilgalys 2003; Guerra-García et al. 2008; Kholia and Fraser-Jenkins 2011; Shea et al. 2011). This situation renders Tenebrionidae collections of little use for any scientific purpose, as it can be easily recognised by their poor representation in biodiversity databases (e.g., GBIF – Gaiji et al. 2013). One example of this problem is represented by the saproxylic hunchback darkling beetles of the genus *Misolampus* Latreille, 1807, paradoxically one of the better studied genera of Tenebrionidae at taxonomic and phylogenetic levels in the Western Palearctic Region (Palmer 1998; Palmer and Cambefort 2000).

The genus *Misolampus* [type species: *Misolampus hoffmannseggii* Latreille, 1807 (= *Pimelia gibbula* Herbst, 1799), by monotypy], currently included within Cnodalonini Oken, 1843, in the subfamily Stenochiinae Kirby, 1837 (= Coelometopinae Schaum, 1859; = Cnodaloninae; see Bouchard et al. 2005, 2011), encompasses six species: *M. gibbulus* (Herbst 1799), *M. goudotii* Guérin-Méneville, 1834, *M. lusitanicus* Brême, 1842, *M. ramburii* Brême, 1842, *M. scabricollis* Graells, 1849, and *M. subglaber* Rosenhauer, 1856 (Löbl et al. 2008; Martínez Fernández 2018), all of them linked to woodlands (Español 1949, 1954b; Molino Olmedo 1996; Palmer 1998). Five of them are endemic to the Iberian Peninsula, while *M. goudotii* is distributed throughout Morocco, Algeria and the Balearic Islands (Löbl et al. 2008). Reitter (1917), Antoine (1949, 1954), and Español (1949) provided identification keys for the species of *Misolampus*, and Palmer (1998) illustrated the female genitalia and specific diagnostic characters. Palmer (1998) and Palmer and Cambefort (2000) presented analyses of their geographic distribution and phylogenetic relationships, based on morphological traits. However, a search in the GBIF database (https://www.gbif.org; searched 22-mar-2020), only retrieved a total of 49 records for *Misolampus* (once records for other genera were discarded), three of them identified at genus level, and at least one misidentified species; 14 of 49 had geographic coordinates data, eleven of which corresponded to a single locality. Excluding all additional specimens with imprecise locality data, fewer than 30 specimens remained available for scientific use.

Despite database records shortfall, the distribution ranges of the species of *Misolampus* are relatively well known (Palmer 1998). Nevertheless, a few new eccentric geographical records have recently been published, suggesting that the distribution areas might be larger than what is currently recognised (Ibáñez Orrico 2002; Pérez and López-Colón 2010; Novoa et al. 2014). Regretfully, many of the specimens used to delineate the distribution areas of the species were collected between 50 and 100 years ago (Palmer 1998). The continuity of those populations through time, under the current scenario of drastic increase in land-use and climate change is, however, questionable (Vanwalleghem et al. 2017), all the more so given the saproxylic nature of these species, which often can lead to conservation issues (García-López et al. 2016).

In the light of these considerations, first, we aimed to provide an updated geographic distribution range for all the species of *Misolampus*, to evaluate their persistence in the areas where they were reported. For this purpose, we undertook a thorough bibliographical revision, an exhaustive field search, and we revised the *Misolampus* collection of the Museo Nacional de Ciencias Naturales (MNCN-CSIC) in Madrid (Spain). Secondly, and as a result of the field data collection, we aimed to determine the taxonomic status of geographically isolated populations of the genus, including those that have been the subject of taxonomic controversy (Reitter 1917; Español 1949; París García et al. 2011). Thirdly, with all that information, we aimed to provide an easy to use, photographically illustrated catalogue for *Misolampus*, and to discuss the potential threats and conservation status of the species of the genus.

## Materials and methods

Field work to locate *Misolampus* was carried out by members of the research team for two periods, a non-intensive period from 1982 to 2000 in which specimens were collected, georeferenced, and dry-mounted for their morphological study, and a more intensive period from 2001 to 2013, with additional collections in 2019–2020, aimed to detect changes in populations previously known from records dating from the 19^th^ and 20^th^ centuries. Field data collection was carried out along most of the areas where the presence of the genus was documented (Spain, Portugal, and Morocco). Information on the location of previously known populations was obtained by undertaking an exhaustive bibliographic revision and by reviewing the *Misolampus* collection held at the MNCN (Museo Nacional de Ciencias Naturales, Madrid, Spain).

We studied 1304 specimens representing all known taxa of *Misolampus* (812 collected before 1945, and 492 collected after 1982). Of those, 355 specimens are preserved in ethanol, and 949 specimens dry-mounted (Table 1), all forming part of the entomological collections of the Museo Nacional de Ciencias Naturales (MNCN-CSIC, Madrid). The list of examined specimens is included in the corresponding paragraph of the species catalogue. Collectors are specified when different from authors or members of the research team; collector name is indicated for old collections only when printed in the labels; “ex.” or “exx.” is used to abbreviate “specimen” or “specimens”.

**Table 1.** Specimens of *Misolampus* studied. Number of specimens by preservation mode (ethanol or dry-mounted) and date of collection (before 1945 or after 1982). The total number of specimens of each species is also provided.

Species	Before 1945	After 1982	Dry-mounted	Alcohol	Total
*Misolampus gibbulus*	339	163	392	110	502
*Misolampus goudotii*	108	66	145	29	174
*Misolampus lusitanicus*	1	27	1	27	28
*Misolampus ramburii*	11	26	32	5	37
*Misolampus scabricollis*	310	178	328	160	488
*Misolampus subglaber*	43	32	51	24	75

Unresolved taxonomic issues, such as the validity of subspecies within the North African taxon, the specific assignation of the populations from Algarve (Reitter 1917; Español 1949), and taxonomic status of the isolated population from Ifni (Morocco) (París García et al. 2011), were addressed by comparing these problematic populations with specimens from near type localities, or from areas of undisputed taxonomy.

Distribution maps based on current data represent the extent of occurrence of each species following a relaxed modification of IUCN criteria (IUCN 2012). We performed species distribution models (SDMs) to obtain the potential distribution of each species (Kamiński et al. 2017). We used Maximum entropy algorithm (MaxEnt) (Elith et al. 2006, 2011) and the set of WorldClim v 2.0 environmental variables, with a resolution of 30 s (~ 1 km) (Fick and Hijmans 2017). The SDMs were modelled considering the studied specimens as presences and generating pseudo-absences following Gil-Tapetado et al. (2018). This methodology creates a preliminary presence-only coverage model based on the maximum and minimum values of each variable. Areas with environmental values that fall out of the maximum and minimum range were considered liable to be pseudo-absences. This is considered as a more reliable approach than generating pseudo-absences entirely at random. SDMs were run 50 times, with random test percentage set to 25 and “subsample” as the sampling technique. The model was validated by estimating the area under the curve (AUC) value (Fielding and Bell 1997). All SDMs have AUC > 0.95 (*M. gibbulus*: 0.958; *M. goudotii*: 0.965; *M. lusitanicus*: 0.998; *M. ramburii*: 0.963; *M. scabricollis*: 0.964; *M. subglaber*: 0.958).

To obtain morphological data, dry-mounted specimens were examined under a stereomicroscopy. Specimen length was measured in dorsal view as the distance between the anterior margin of the pronotum and the elytral apex (ignoring elytral convexity). The head was excluded from measurement since it is usually directed ventrally. Maximum width was measured as the distance between the outer edges of the elytra at approximately three-fourths of the elytral length, also in dorsal view. Photographs of live specimens were taken with a Nikon digital camera. Extended depth-of-focus images of dry-mounted specimens, were taken on a Leica M165C stereo-microscope, with a digital camera Leica DFC450, using the LAS X software from Leica Microsystems.

## Results

### Species catalogue, distribution, notes on natural history, and taxonomy

#### Misolampus gibbulus

Taxon classificationAnimaliaColeopteraTenebrionidae

(Herbst, 1799)

DE0F63A3-C070-50F4-AB22-B336FBB55932

Pimelia gibbula Herbst, 1799: 51. Terra typica: unknown: “Das vaterland ist mir unbekannt”.
Misolampus hoffmannsegii Latreille, 1807: 161. Terra typica: “e Lusitania allatus”. Latreille’s (1807) species name has been often misspelled. Guérin-Méneville (1829–1838: 115, pl. 29; 1834: 27) spelled it as: “M. hoffmansegii” and “M. hoffmanseggii”, respectively, Solier (1848: 185): “M. hoffmanseggii”, and Laporte de Castelnau (1850: 204): “M. hoffmansseggii”. Synonymy with M. gibbulus proposed by Solier (1848).
Misolampus gibbulus (Herbst, 1799): Solier 1848: 185.

##### Studied material.

Portugal – Beja: Beja: 1 ex.; Beja, V-1909 (exp. del Museo): 1 ex.; São Martinho das Amoreiras, 200 m, 37°36'57.4"N, 08°27'57.3"W, 4-I-2013: 10 exx. – Evora: Evoramonte, 17-X-1992: 1 ex.; Monte São Bento, 353 m, 38°34'54.33"N, 7°56'12.10"W, 11-III-2010: 1 ex.; Valverde, 232 m, 38°31'39.8"N, 8°00'25.4"W, 4-X-2002: 1 ex.; – Faro: Alferce: 1 ex.; Alferce, V-1909 (Exp. del Museo): 2 exx.; carretera Monchique-Laranjeira [Gil Bordalo], 21-X-1992: 6 exx.; Foia, 742 m, 37°18'29.4"N, 08°35'56.2"W, 4-I-2013: 1 ex.; Monchique, 439 m, 37°21'40.3"N, 08°32'23.6"W, 4-I-2013: 8 exx.; San Marcos da Serra [São Marcos da Serra]: 2 exx.; San Marcos da Serra [São Marcos da Serra], V-1909 (exp. del Museo): 23 exx.; São Marcos da Serra, 140 m, 37°21'02.5"N, 08°22'48.4"W, 3-I-2013: 24 exx.; Sierra de Monchique, V-1909 (exp. del Museo): 1 ex.; Portalegre: Santo Antonio de Alcorrego, 150 m, 38°58'59.5"N, 7°56'54.1"W, 18-IV-2013: 6 exx. Spain – Andalucía: Córdoba: Córdoba (Col. del Sr. Pérez Arcas): 2 exx.; Córdoba: 2 exx.; Córdoba, IV-1901 (Escalera leg.): 4 exx.; Córdoba, VI-1909 (Exp. del Museo): 3 exx.; Manueles, 30SUH82, 7-V-1982 (M.A. Alonso Z. leg.): 1 ex.; Huelva: Barranco Riofrío [La Nava], 28-XII-1985: 1 ex.; Cala (C. Bolívar leg.): 15 exx.; Cortegana, Puerto del Corzo (hacia Gil Márquez), 664 m, 37°52'56.1"N, 06°50'42.3"W, 3-I-2013: 7 exx.; Patrás, 397 m, 37°48'04.4"N, 6°43'30.8"W, 1-V-2004: 3 exx.; Jaén: [3 km al SO de] Aldeaquemada, 25-IV-1992: 3 exx.; Lugar Nuevo, 24-X-1991: 2 exx.; Santa Elena, carretera hacia La Aliseda, 795 m, 38°20'53.1"N, 03°33'20.6"W, 28-XII-2010: 14 exx.; Santa Elena, 12-III-1901: 2 exx.; Santa Elena: 2 exx.; Sierra Morena: 1 ex.; Sevilla: Constantina: 1 ex.; – Castilla –La Mancha: Ciudad Real: Almadén (Belbeze leg.): 1 ex.; Navas de Estena: 1 ex.; Pueblo Nuevo del Bullaque, 7-XII-1992: 2 exx.; Puerto Madrona, 20-XI-1992: 8 exx.; Saceruela (Paz leg.): 1 ex.; Solana del Pino: Puerto Madrona, 38°25'07.3"N, 4°03'33.1"W, 06-III-2012: 1 ex.; Toledo: Santa Cruz del R. [Retamar] (Paz leg.) (Col. del Sr. Pérez Arcas): 1 ex.; – Castilla y León: Ávila: Candeleda, XI-1933: 1 ex.; 8 km NE Hoyo de Pinares, 40°31'40.6"N, 4°20'04.5"W, 1-IV-2013: 2 exx.; Mombeltrán – Navalperal [de Pinares]: 1 ex.; Extremadura: Badajoz: Aljucén (Pacheco leg.): 2 exx.; Cáceres: Alcuéscar: I-1894: 3 exx.; Belvís de Monroy, 373 m, 39°48'04.8"N, 5°37'01.1"W, 24-XII-2011: 6 exx.; Castillo de Trevejo, 714 m, 40°10'20.9"N, 6°46'48.9"W, 17-IV-2011: 1 ex.; Valdemorales, 420 m, 39°12'08.1"N, 06°03'57.8"W, 2-I-2012: 2 exx. Madrid: Brunete (Bolívar leg.): 2 exx.; Cadalso [de los Vidrios] (J. Ardois leg.): 120 exx.; Cadalso de los Vidrios, hacia Almorox, 12-IV-1992: 1 ex.; Cerro de San Pedro, 29-X-2004: 4 exx.; Collado Mediano (C. Bolívar leg.): 1 ex.; Collado Mediano: 13 exx.; Collado Mediano (G. Schramm leg.): 4 exx.; Collado Mediano (Moróder leg.): 5 exx.; Fresnedillas de la Oliva, 941 m, 40°29'38.57"N, 4°10'12.90"W, 14-III-2001: 14 exx.; Galapagar (Col. del Sr. Pérez Arcas) (*Misolampus gibbulus* Hbst.): 1 ex.; Manzanares [El Real], 30-III-1928: 1 ex.; Moralzarzal: Cerro del Telégrafo, 23-IV-2017: 1 ex.; Navas del Rey, 2-XII-1990: 2 exx.; Pelayos de la Presa, 799 m, 40°20'19.40"N, 4°21'34.84"W, 3-III-2001: 1 ex.; [3 km al S de] Quijorna, 5-II-1992: 1 ex.; Robledo de Chavela: 7 exx.; Santa María de la Alameda (estación), 1-IV-1991: 10 exx.; Sierra de Guadarrama (J. Lauffer leg.): 1 ex.; Torrelodones, 7-XI-1992: 4 exx.; Valdemaqueda, 40°30'30.0"N, 4°17'00.1"W, 1-IV-2013: 2 exx.; Valdemorillo, 12-IV-1992: 7 exx.; Villa del Prado (J. Ardois leg.): 105 exx.; Villa del Prado: 4 exx.; Villa del Prado, Encinar del Alberche, 742 m, 40°17'29.7"N, 04°21'11.9"W, 4-I-2009: 5 exx.; Villalba: 1 ex.

##### Diagnosis.

**Figure 1.**
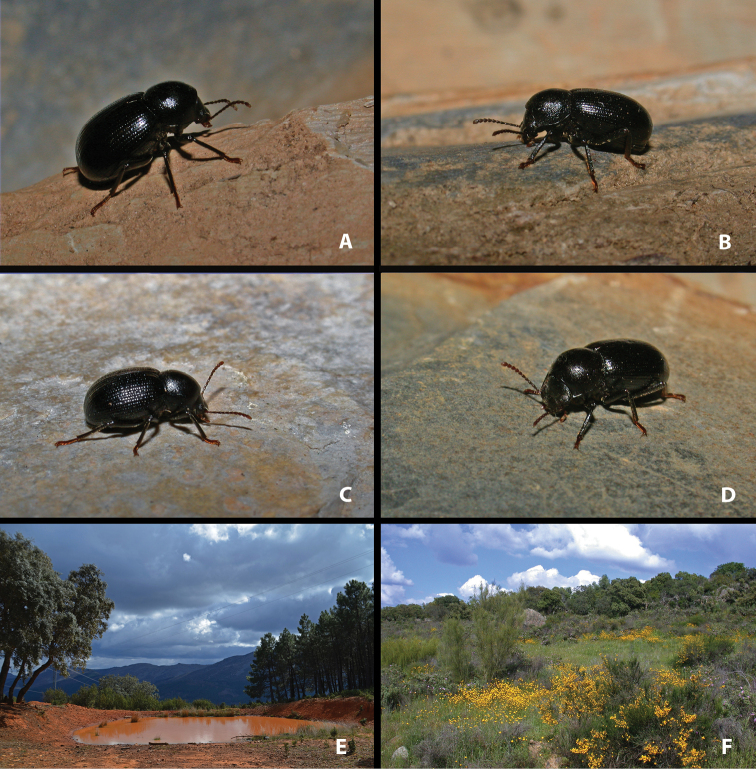
Live specimens and habitat of *Misolampus gibbulus***A–D** live adult specimens of *Misolampus gibbulus* from Portugal (**A** Foia; **B** Monchique; **D** São Martinho das Amoreiras) and Spain (**C** Santa Helena, Jaén); specimens **A**, **B**, and **D** represent the diversity of sculptural patterns in elytra and pronotum within the Faro population, see the contrast with typical specimen **C**; **E, F** typical habitats of *M. gibbulus* in Spain (**E** native *Quercus ilex* and *Pinus* plantations at Robledo del Mazo, Toledo **F***Q. ilex* open forest with *Cytisus* and *Retama* at Puerto de Santa Cruz, Cáceres). Photographs by MGP.

Total length 6.6–12 mm (Reitter 1917; Español 1949; López-Pérez 2014a). Easily recognisable by its general shiny appearance and small size. *Misolampus gibbulus* presents acutely protruding prothoracic anterior angles, strong pronotal punctation, deep, and densely covering most of its surface; elytra with well-marked deeply excavated striae, with large and deep punctation, and shiny interstriae intervals often with additional series of punctures (Fig. 1A–D). Female genitalia figured by Palmer (1998). The species presents marked variability on the development and depth of the elytral and pronotal sculpture. Pronotal punctation is usually less developed, and elytral striae shallower, not so excavated, in populations of southwestern Portugal (Faro district) (see taxonomic discussion).

##### Geographic distribution.

**Figure 2.**
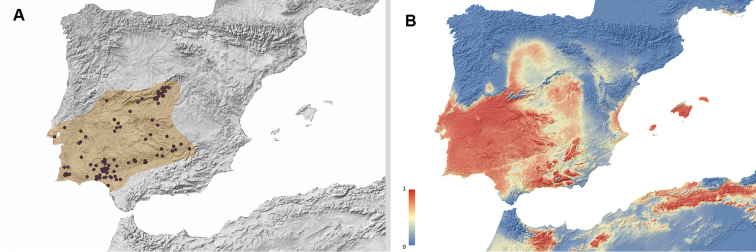
**A** Geographic distribution of *Misolampus gibbulus.* Map of the Iberian Peninsula depicting the geographic distribution range of the Iberian endemic *Misolampus gibbulus* (orange area). Purple dots correspond to the species’ records, including both recent and old, as well as previously published data **B** potential geographic distribution of *Misolampus gibbulus*: Red indicates areas of high suitability, and blue, areas of low suitability. Species distribution model was generated using MaxEnt v 3.4.1 (Elith et al. 2006) and the set of WorldClim v 2.0 (Fick and Hijmans 2017) environmental variables.

Endemic to Spain and Portugal (Löbl et al. 2008) (Fig. 2). Its general distribution includes most of the southwestern area of the Iberian Peninsula. Published records are however scanty, from central and southern Portugal, and from the Spanish provinces of Cáceres, Ciudad Real, Córdoba, Huelva, Jaén, Madrid and Sevilla (Latreille 1807; von Heyden 1870; Paulino de Oliveira 1894; Reitter 1917; Lindberg 1933; De la Fuente 1934–1935; Español 1949; Cabral 1983; Cárdenas Talaverón and Bujalance de Miguel 1985; Cárdenas 2003; Grimm and Aistleitner 2009; López-Pérez 2014a, 2014c; Bujalance de Miguel 2015; Barreda 2018).

Our new records considerably expand the known distribution of *M. gibbulus*. In addition to previously published data, we add new records for the district of Évora and Portalegre in Portugal, and from the provinces of Ávila, Badajoz, and Toledo in Spain; together with numerous localities for some provinces represented by a few records, such as Cáceres, Ciudad Real, and Madrid. With the addition of these records, the distribution of *M. gibbulus* seems to be more or less continuous along the southern slopes of the Sistema Central: from Cáceres and Ávila to Madrid, along both slopes of Montes de Toledo and Sierra Morena, and in a more or less extended area in southern Portugal, from Évora to Serra de Monchique in the Algarve region. The Guadalquivir river basin seems to conform the southeastern distribution limit for the species (Fig. 2A). The potential distribution map identifies southwestern Iberia as a high suitable area for the species occurrence, together with some areas where the species does not occur: the Betic Mountain ranges, the Balearic Islands, and northern Africa (Fig. 2B).

##### Notes on natural history.

*Misolampus gibbulus* is a low altitude species, ranging from 4 to 1278 m a.s.l., although 81% of the populations recorded are located below 800 m of altitude. Geological substrates are very diverse across its distribution area, but mostly siliceous, including sandstones, gneisses, granites, and schists, which generate acid soils (see Vera 2004; Oliveira and Quesada 2019a, 2019b). It occupies mainly the meso-Mediterranean thermoclimatic belt and, to a lesser extent, the thermo – (at the southermost portion of its range) and supra-Mediterranean (on a narrow northern strip), with ombrotypes from dry to humid (Rivas-Martínez 1987; Rivas-Martínez et al. 2002; Rivas-Martínez 2007). It is found over an extensive variety of forest and subforestry habitats, including both coniferous (*Pinus* L.) and broadleaved trees (*Quercus* L., *Fraxinus* L.), and also dense shrublands of *Cistus* L. (“jarales”), *Retama* Raf. and *Cytisus* Desf. (“retamares”) (Fig. 1E, F). The species also occupies areas densely reforested with native and non-native *Pinus* and *Eucalyptus* L’Hér. (Cabral 1983), as well as open man-modified agroforestry systems (“dehesas” of *Quercus*) and montane agrosystems with olive and chestnuts trees (*Olea europaea* L. and *Castanea sativa* Mill.) (see Ladero 1987; Rivas-Martínez et al. 1987; Valle 2003; Costa Tenorio et al. 2005).

*Misolampus gibbulus* is commonly found under bark or within decomposing dead logs and stumps of pines (mainly of *Pinus pinea* L. and *Pinus sylvestris* L.), including reforested areas (especially *Pinus pinaster* Aiton), where they appear to be particularly common. It is also found in dead or old trunks of perennial or deciduous oaks (*Quercus ilex* L., *Quercus suber* L., *Quercus pyrenaica* Willd. and *Quercus faginea* Lam.), under the dry layers that cover roots and thick stems of *Cistus ladanifer* L. and *Cistus laurifolius* L., and at the base of brooms, mainly *Cytisus scoparius* (L.) Link and *Retama sphaerocarpa* (L.) Boiss. Occasionally found under loose bark or at the base, among decaying wood of standing *Eucalyptus* trees, and also in rotten *Eucalyptus* stumps (Cabral 1983; López-Pérez 2014a; pers. obs.). Sometimes found also under stones in open areas, near forest or shrub patches. Almost all these habitat locations are coincident to those described by López-Pérez (2014a) for the province of Huelva. Its food source is unknown (as in the other species of the genus), although Barreda (2018) pointed out mistakenly that it is a moss eater (quoting Español 1949 and Bujalance de Miguel 2015); nevertheless, Español (1954b) commented that the species of *Misolampus* are saprophagous, without further specification.

*Misolampus gibbulus* has been found in microsympatry with *M. scabricollis* along western Sierra Morena (Huelva), northern Extremadura (Cáceres), Montes de Toledo (Toledo) (Fig. 1E), and southern slopes of the Sistema Central (Madrid, Ávila, Toledo), and with *M. subglaber* at the eastern end of Sierra Morena (Jaén) (pers. obs.). Adults can be found across most of the year (Cárdenas Talaverón and Bujalance de Miguel 1985; López-Pérez 2014a; Barreda 2018) but according to our observations they are more easily encountered during the wetter months (October to May).

#### Misolampus goudotii

Taxon classificationAnimaliaColeopteraTenebrionidae

Guérin-Méneville, 1834

AF57D823-BE12-5C5D-B5EF-0C611A597DA3

Misolampus goudotii Guérin-Méneville, 1834: 28. Terra typica: “trouvée à Tanger... ...à trois lieues de Tanger, sur les bords d’une rivière, dans le tronc d’un olivier.” Vauloger de Beaupré (1900), Reitter (1917), Antoine (1949), and Español (1949) among others, wrote the species name with a single final -i. Solier (1848) and Vauloger de Beaupré (1900) mentioned the unavailable name: “Misolampus nigrita Dejean”, and Español (1954a): “M. moraguesi”.
Misolampus goudotii Erichson in Wagner, 1841: 184 (non Guérin-Méneville 1834). Terra typica: not indicated, but “von Algier” according to Erichson’s (1841) work title.
Misolampus erichsoni Vauloger de Beaupré, 1900: 674 syn. nov. Terra typica: “Algérie: O., Oran...; Daya...; Tlemcen...; Mascara..., Ammi Moussa; A.: Blidah...; La Chiffa...; Margueritte...; forêt de Boghar...; mont Ouarsenis...; forêts de la Grande-Kabylie...”.
Misolampus peyerimhoffi Antoine, 1926: 257 syn. nov. Terra typica: “Grand Atlas, région du Glaoui: plateau des Aït Rba...”.

##### Studied material.

Algeria: “Argelia” (Dufour leg.): 1 ex. Morocco – Marrakech-Tensift-Al Hauz: Toufliht, 1483 m, 31°28'34.6"N, 7°26'06.5"W, 11-III-2013: 4 exx. – Meknès – Tafilalet: Ain Leuh, 17-V-1925: 1 ex.; Azrou, 1900 m (Alluaud 79) (*Misolampus goudoti var. laevior* Alluaud): 1 ex.; Azrou, 19-V-1925: 1 ex.; Dj. [Yebel] Hebri, 20-V-1925: 1 ex.; Timadit [Timahdite], 21-V-1923: 1 ex. – Tanger – Tétouan: Rif: Beni Siyyel: Bab Ruadi: VI-1932 (C. Bolívar leg.): 6 exx.; Tanger, 1897: 3 exx.; Tanger (M. Escalera leg.) (small square-label pinned): 36 exx., plus 3 exx. only square-labelled; 2 km al O de Bab Berret, 1318 m, 35°00'02.57"N, 4°55'31.91"W, 12-VI-2011: 3 exx.; Crtra. Zinat-Mulay Abdeselam, P-4702, Beni Aros, Yebala, 513 m, 35°22'04"N, 5°32'17"W, 29-IV-2016: 5 exx.; Yebel Bou-Hachem, Beni Aros, Yebala, 1160 m, 35°15'31"N, 5°30'49"W, 12-V-2012: 6 exx.; Crtra. Mulay Abdeselam-Al Hamra, P-4704, Beni Aros, 985 m, 35°15'50"N, 5°25'36"W, 28-XI-2019: 2 exx.; Larache: Yebala: Beni Arós: Yebel Bou-Hachem, 35°15'31"N, 5°30'49"W, 9-VI-2012: 2 exx.; Pinsapar del Talassemtane, 1900 m, 35°08'36.7"N, 5°08'13.0"W, 11-VI-2011: 2 exx.; Bab Taza: Talassemtane, 1401 m, 35°06'10.9"N, 5°08'21.3"W, 27-VII-2013: 1 ex.; Bab Taza: Talassemtane: Plaza de España, 1667 m, 35°09'03.7"N, 5°08'28.6"W, 27-VII-2013: 1 ex.; Casa Forestal, Yebel Lekraa, P.N. Talasemtane, Chefchaouen, 35°07'56"N, 5°08'11"W, 1695 m, 7-VI-2008: 3 exx.; Yebel Talassemtane-vertiente sur, P.N. Talasemtane, Chefchaouen, 35°07'53"N, 5°08'01"W, 1650 m, 11-IV-2011: 4 exx.; P.N. Yebel Tazaot, Pinsapar, P.N. Talasemtane, Chefchaouen, 35°15'N, 5°07'W, 1670 m, 7-V-2011: 2 exx.; Pista hacia Casa Forestal, Yebel Lekraa, P.N. Talasemtane, Chefchaouen, 35°07'45"N, 5°08'09"W, 1700 m, 28-VII-2011: 1 ex.; E. de Yebel Talaousisse, P.N. Talasemtane, Chefchaouen, 35°07'33"N, 5°04'03"W, 1350 m, 1-XII-2018: 2 exx.; Pista hacia Haout Taznout, P.N. Talasemtane, Chefchaouen, 35°08'20"N, 5°07'24"W, 1712 m, 27-IV-2019: 2 exx.; Yebel Tizirhen, Bab Berred, Rif Central, 1585 m, 35°00'54"N, 4°54'57"W, 27-IV-2017: 3 exx.; Yebel Tizirhen, Bab Berred, Rif Central, 1570 m, 35°00'47"N, 4°54'03"W, 28-IV-2018: 1 ex.; Pista de Bab El Kar, Montañas de Fifi, Rif, 1512 m, 34°59'13"N, 5°11'20"W, 2-VI-2019: 2 exx. – Taza – Al Hoceima – Taounate: Iguermalen [Targuist]: Beni Mesdui, VI-1932 (M. Escalera leg.): 6 exx.; Rif: Beni Seddat: Imosiner: VI-1930 (exp. C. Bolívar leg.): 3 exx.; Rif: Beni Seddat: Tizi Taka, VI-1932 (C. Bolívar leg.): 4 exx.; Rif: Beni Seddat: Tizi Taka, VI-1932 (Exp. C. Bolívar leg.): 1 ex.; Rif: Iguermalen (Targuist), VI-1930 (exp. C. Bolívar leg.): 4 exx.; Rif: Ketama, Bab Chiquer, VI-1932 (C. Bolívar leg.): 8 exx.; Rif: Ketama, Bab Chiquer, VI-1932 (M. Escalera leg.): 2 exx.; Rif: Ketama: Tainza, VI-1930 (exp. C. Bolívar leg): 3 exx.; Rif: Ketama: Tidiguin [Tidghine], VI-1930 (exp. C. Bolívar leg.): 1 ex.; Rif: Ketama: Zoco Telata, VI-1932 (M. Escalera leg.): 7 exx.; Lurdeka [?]: 1 ex.; Yebel Tidighin, Azila, Rif central, 1705 m, 34°51'14"N, 4°32'19"W, 29-XI-2019: 1 ex; Carretera P-5420, P.N. Tazzeka, Medio Atlas nororiental, 1000 m, 34°03'N, 4°15'W, 25-XI-2004 (F.J. Martínez leg.): 2 exx. – Souss-Massa-Drâa: Yebel Tual, 28-VII-1934: 1 ex.; Ifni: Yebel Tamarrut [25 km SE Ifni], I-1935 (F. Escalera leg.): 1 ex.; Sidi Ifni: Akarkor, Jbel Toual, 627 m, 29°13'48.9"N, 10°00'44.1"W, 21-I-2020: 4 exx. Spain – Islas Baleares: Mallorca (Mas de Xaxars leg.) (*Misolampus erichsoni*): 2 exx.; Escorca, 26-III-1985, 1 ex.; Escorca, Coll de Femenia, 545 m, 39°51'33.68"N, 2°54'19.27"W, 25-III-2012: 10 exx.; Menorca (Cardona leg.): 2 exx. plus 6 exx. without data; 2 exx.; Menorca: 2 exx.; Algaiarens, 14 m, 40°02'28.3"N, 03°55'28.4"W, 27-IV-2006: 2 exx.

##### Diagnosis.

**Figure 3.**
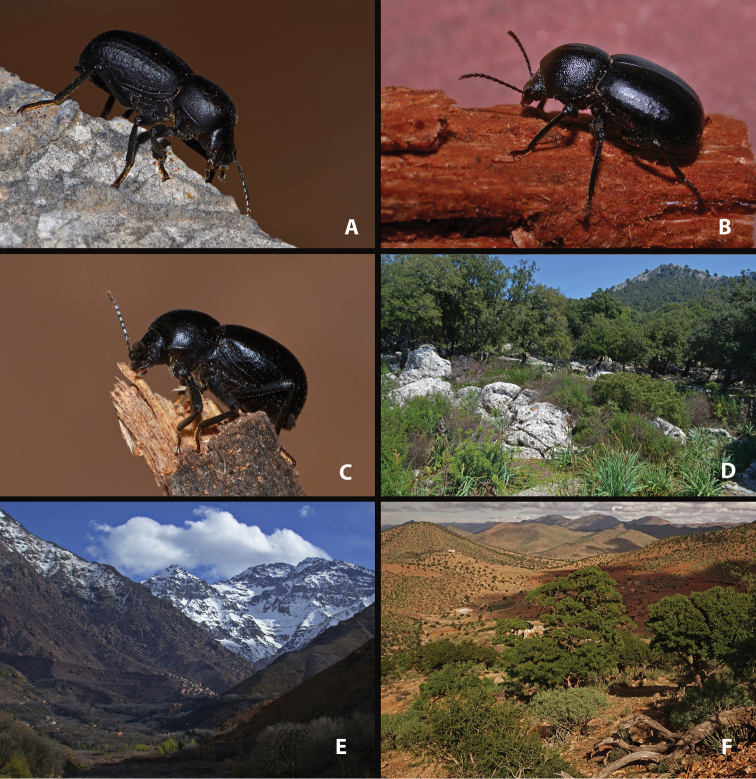
Live specimens and habitat of *Misolampus goudotii***A–C** Live adult specimens of *Misolampus goudotii* from the Balearic Islands (**A** Cap Formentor, Mallorca) and Morocco (**B** Toufliht, High Atlas **C** Akarkor, Sidi Ifni); the specimens selected represent the diversity of sculptural patterns in elytra and pronotum reported for the species **D–F** A summary of the impressive habitat diversity used by *M. goudotii* from the Balearic Islands (**D***Quercus ilex* forest at Creu de Menut, Mallorca), to southwestern Morocco (**E** deep valleys in the Toubkal National Park, High Atlas **F***Argania spinosa* open forests at Jbel Toual in Sidi Ifni). Photographs by MGP and NRR.

With a total length from 10 to 14 mm, this is the largest species of the genus (Vauloger de Beaupré 1900; Reitter 1917; Antoine 1926; Español 1949). This species is well characterised and isolated within the genus *Misolampus* by the following traits: fore angles of the prothorax not protruding, almost rounded, forming an obtuse angle at apex; lateral surface of pronotum shallowly rugose, with the rugosity progressively erased towards the dorsal areas that appear smoother, propleural punctation fine and often erased; elytra with longitudinal series of small elongated tubercles, more apparent on the sides of the posterior half of the elytra (Español 1949, 1954a; Palmer 1998) (Fig. 3A–C). Female genitalia figured by Palmer (1998). Specimens from the Balearic Islands have been studied karyologically (Juan and Petipierre 1986, 1989; Juan et al. 1993; Pons et al. 1993; Pons 2004), presenting a chromosome number of 20 (2n) (Juan and Petitpierre 1986, 1989). There is marked geographical variability on the sculpture and shape of pronotum and propleurae, and on the development of elytral tubercles (Vauloger de Beaupré 1900; Antoine 1949; Español 1954a) (Fig. 3A–C). Specimens from northern Morocco (excluding the Tingitane Peninsula), Algeria and the Balearic Islands, present a well-developed and evident elytral tuberculation that may form rugose ridges (Fig. 3A). On the other extreme, elytral tubercles are reduced in the Rif and Atlas populations (Fig. 3B), to become almost completely absent in the specimens from Sidi Ifni (Fig. 3C). Pronotum sculpture is formed by fine spaced punctures intermixed with granules, much denser on the sides in the Balearic Islands population (Fig. 3A); pronotal rugose areas are more marked and extended in the specimens from the High Atlas (Fig. 3B), and formed by sparse punctation, without granulose areas, in the specimens from Ifni (Fig. 3C). The anterior edge of the pronotum, in the Rif and Balearic populations, is straight at the middle, while it appears convex in the populations from the High Atlas (Antoine 1949). The geographic distribution of this variability has been the subject of taxonomic discussion resulting in the proposal of different taxa, here formally synonymised (see synonymic list, and taxonomic discussion).

##### Geographic distribution.

**Figure 4.**
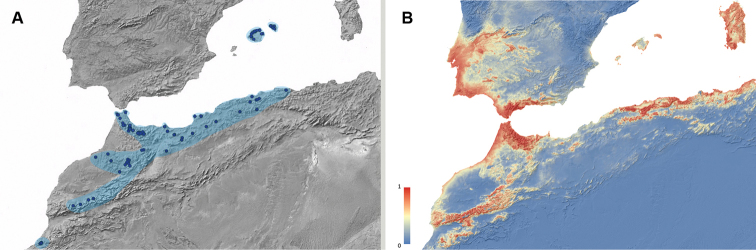
**A** Geographic distribution of *Misolampus goudotii*. Map for the distribution range of *Misolampus goudotii* (pale blue spot). Blue dots correspond to the species records, including both recent and old, as well as previously published data. The population from Ifni remains isolated from the main distribution range, by a distance of ca. 250 km **B** potential geographic distribution of *Misolampus goudotii*: Red indicates high suitable areas, and blue, areas of low suitability. Species distribution model was generated using MaxEnt v 3.4.1 (Elith et al. 2006) and the set of WorldClim v 2.0 (Fick and Hijmans 2017) environmental variables.

Distributed throughout Morocco, northern Algeria and the Balearic Islands in Spain (Antoine 1926, 1949; Español 1949; Löbl et al. 2008). Precise records are well distributed in northern Morocco and Mallorca, scanty in all other areas (Solier 1848; Lucas 1849; Cardona Orfila 1875; Pérez Arcas 1873; Moragues 1889; Champion 1891; Vauloger de Beaupré 1900; Martínez de la Escalera 1914; Reitter 1917; Peyerimhoff 1919; Antoine 1926; Lindberg 1933; De la Fuente 1934–1935; Palau 1945; Antoine 1949; Español 1949, 1953, 1954a; Cobos 1955; Pardo Alcaide 1955; Kocher 1958; Cobos 1961; Español 1967; Mouna and Arahou 1986; Juan and Petitpierre 1989; Whitehead 1993; París García et al. 2011; Benyahia et al. 2015, 2016; Núñez et al. 2016; Chavanon 2020) (Fig. 4). The record from Ceuta, Spain (Vauloger de Beaupré 1900) corresponds to the mountain Yebel Musa (just 1.5 km west of Ceuta), currently in Moroccan territory (region of Tanger-Tétouan).

The studied materials include recent and old records of populations from the Balearic Islands (Mallorca and Menorca) and from the Moroccan regions of Meknès-Tafilalet, Souss-Massa-Drâa, Tanger-Tétouan, and Taza-Al Hoceima-Taounate. Recent data are available from all four regions, with a large number of localities from the Rif, and less numerous in the Middle and High Atlas. Among these records, we emphasise the re-discovery of the population from the province of Sidi Ifni, in January-2020, 85 years after its original finding, by F. Martínez de la Escalera in 1934 and 1935 (París García et al. 2011). The latter is a singular population, apparently isolated in the arid mountains near Ifni; its closest known population is located in the Western High Atlas, ca. 250 km to the northeast (Fig. 4A). The potential distribution map locates high suitable areas for this species along the mountain ranges of northwestern Africa, the coastal and mountain areas in the Tingitane peninsula, and along the coast of Rabat-Salé-Kénitra region. It also identifies areas where the species does not occur as high suitable, including sothwestern Iberia, the Balearic Islands and Sardinia. The Ifni population is located in a very fragmented area of high suitability, suggesting a possible Pleistocene relict status for this population (Fig. 4B).

##### Notes on natural history.

*Misolampus goudotii* is widely distributed over northwestern Africa, though restricted to mountain ranges and adjacent areas: Rif, Middle Atlas, western High Atlas, Beni Snassen mountains, southwestern foothills of the Anti-Atlas (Morocco) and Tellian Atlas (Algerie) (Fig. 4). Altitudinal range in the Maghreb from 2 to 2064 m a.s.l., with 70.5% of records above 800 m of altitude (62% above 1000 m). In the Balearic Islands its altitudinal range is lower, between 15 and 718 m a.s.l., but the species is found mainly in areas of mountainous topography (e.g., Serra de Tramuntana in Mallorca). It inhabits a wide range of geological substrates, both acid and basic, from plutonic and metamorphic types to calcareous and dolomitic rocks (see Michard 1976; Vera 2004; Oliveira and Quesada 2019a, 2019b). *Misolampus goudotii* is a euryecious species that occurs at infra-, thermo-, meso- and supra-Mediterranean thermoclimatic belts, in regions with ombrotypes from arid to hyperhumid (Benabid 1985; Rivas-Martínez 1987; Le Houerou 1989; Rivas-Martínez et al. 2002; Rivas-Martínez 2007; Sebbar et al. 2013), and occupies a wide variety of forest formations, both coniferous [*Tetraclinis articulata* (Vahl) Mast., *Abies maroccana* Trab., *Cedrus atlantica* (Endl.) Manetti ex Carrière, *Juniperus phoenicea* L., *Juniperus thurifera* L., *Pinus nigra* J.F. Arnold, *Pinus halepensis* Mill., *P. pinaster*] and broadleaved [deciduous: *Quercus canariensis* Willd., *Quercus afares* Pomel, *Q. faginea*, *Q. pyrenaica*; perennial: *Quercus ilex*, *Q. suber*, Olea europaea var. sylvestris (Mill.) Lehr] (see Benabid 1982, 1984, 1985; Benabid and Fennane 1994; Bolòs 1997; Charco 1999; Benabid 2000; Taleb and Fennane 2019). It also occurs in areas reforested with pines (pers. obs.) (Fig. 3D, E). The population of Ifni inhabits mountains (620–1225 m of altitude) at the infra-Mediterranean thermoclimatic belt, probably affected by the proximity to the Atlantic Ocean and consequently by the presence of some degree of cryptic precipitation (Géhu and Biondi 1998). The vegetation of the area is dominated by open forest of *Argania spinosa* (L.) Skeels, with sparse cactiform and arbustive *Euphorbia* L. (Médail and Quézel 1999; Ruiz and García-París 2015), and large areas covered by formerly cultivated *Opuntia* Mill (Fig. 3F).

In the Moroccan Rif, *M. goudotii* is often encountered under bark, inside fallen logs or stumps, and at the base of dead old oaks (perennial: *Q. ilex*, *Q. suber*; deciduous: *Q. canariensis*, *Q. faginea* and *Q. pyrenaica*), arbutus trees (*Arbutus unedo* L.), wild olive trees (O. europaea var. sylvestris), pines (*P. nigra*, *P. pinaster*, *P. halepensis*), firs (*Abies maroccana*), and cedars (*Cedrus atlantica*), as already reported partially by Vauloger de Beaupré (1900), Cobos (1955, 1961), and Benyahia et al. (2015, 2016). They can also be found under bark of standing dead trees (*A. maroccana*, *C. atlantica*, *Q. suber*, *Q. pyrenaica*). In the Middle and High Atlas, it is usually found under bark and inside large decaying logs of *Q. ilex* (Antoine 1926), but also in old decomposing logs of *P. nigra* and *C. atlantica*. Mouna and Arahou (1986) collected the species on thuya (*Tetraclinis articulata*) in the Korifla Valley (northwestern Morocco). Sidi Ifni specimens were found within crevices in old dead logs of *Argania spinosa*, almost buried on the ground of a steep slope (Fig. 3F). Nearby standing dead trunks were occupied by *Nesotes tuberculipennis villarubiai* (Español, 1943) as described by Nabozhenko (2015). In Algeria, they have been found under bark of fallen pines (Vauloger de Beaupré 1900). In Menorca, it has been found in oak forests of *Q. ilex*, under bark or under stones and leaf litter (Cardona Orfila 1875), and in Mallorca it is frequent in decaying wood of fallen pines (*P. pinea*) and old oaks (*Q. ilex*) (Fig. 3D).

Adult specimens are often found in aggregations. We found aggregations of approximately 15 specimens close together in a single large rotting pine log in Mallorca. We also found aggregations of *M. goudotii* together with *Helops insignis maroccanus* (Fairmaire, 1873) (Tenebrionidae, Helopinae) under bark of dead trees of *Q. suber*, *A. maroccana* and *C. atlantica* in the Rif Mountains. Whitehead (1993) relates the finding on two occasions of groups of individuals between the annual rings of dead pines (*P. halepensis*) in active colonies of ants of the genus *Messor* Forel, 1890 and of the species *Monomorium bicolor* Emery, 1877 (probably another species of *Monomorium* Mayr, 1855, since the invasive *M. bicolor* is not present in Balearic Islands; Salata et al. 2019).

Adults are present all year round, but they are more commonly seen in winter and spring in middle and low elevations (Vauloger de Beaupré 1900; Español 1967; pers. obs.), and in summer at higher altitude (Antoine 1926), however, Moragues (1889) mentioned collections during the summer in Mallorca.

#### Misolampus lusitanicus

Taxon classificationAnimaliaColeopteraTenebrionidae

Brême, 1842

2F8C3807-BAFC-572B-9B05-A561AE1B8D65

Misolampus lusitanicus Brême, 1842: 82. Terra typica: “Portugal”.

##### Studied material.

Portugal – Porto: Fervença – Eido, 585 m, 41°14'28.98"N, 7°57'00.34"W, 24-IV-2012: 23 exx. Spain – Castilla y León: León: Lago de la Baña, 1418 m, 42°15'23.2"N, 6°44'58.6"W, 22-VIII-2016: 1 ex. – Galicia: Ourense: Fumaces, 804 m, 41°56'50.2"N, 7°21'05.7"W, 20-XI-2012: 3 exx.; Sierra de Oneija [Queixa] (A. Kricheldorff leg.): 1 ex.

##### Diagnosis.

**Figure 5:**
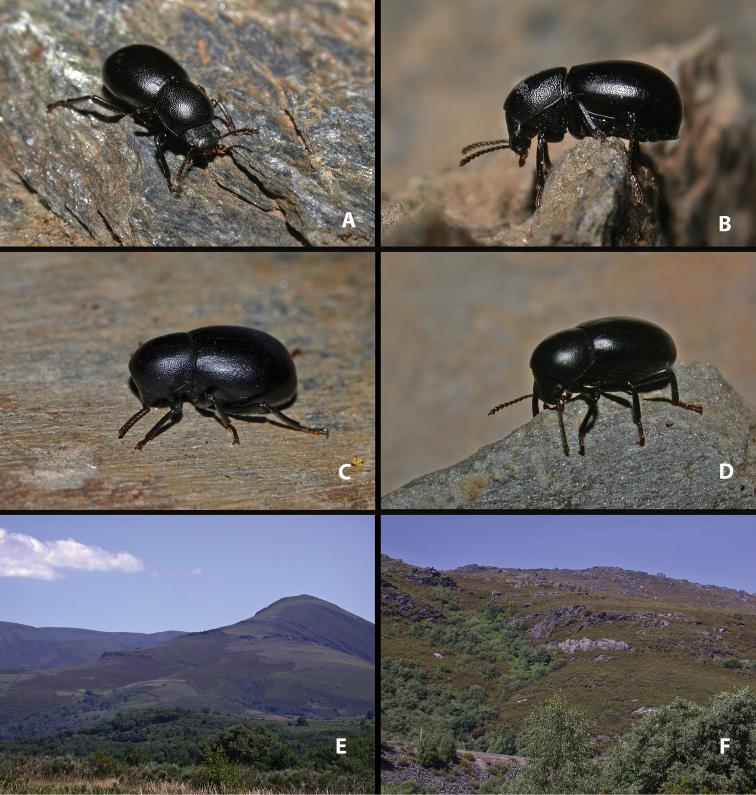
. Live specimens and habitat of *Misolampus lusitanicus***A–D** live specimens of *Misolampus lusitanicus* from Spain (**A, B** Fumaces, Ourense **C** Laguna de La Baña, León) and Portugal (**D** Fervença-Eido, Porto) **E, F** two examples of typical habitat of *M. lusitanicus* from **E** Sierra de Queixa (Ourense) and **F** Mountains of Sanabria (Zamora). Photographs by MGP.

Total length 7.5–8.0 mm, one of the smaller species within the genus (Reitter 1917; Español 1949; pers. obs.). Antennae relatively short, not reaching the base of prothorax (Español 1949). Pronotum with relatively deep, dense, well-defined punctation covering all its surface. Elytra covered by dense punctation somewhat confused with shallow granulation, or partially erased at the disc (Fig. 5A–D). Female genitalia figured by Palmer (1998). We have not observed any relevant morphological variability among the populations studied.

##### Geographic distribution.

**Figure 6.**
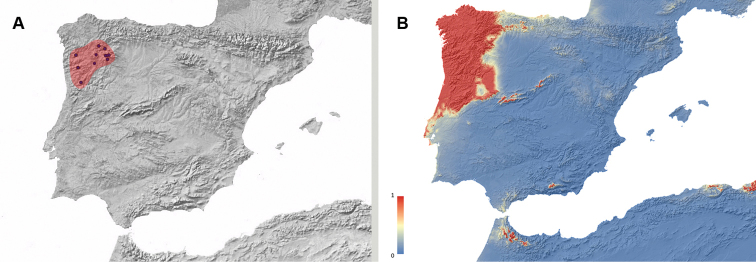
**A** Geographic distribution of *Misolampus lusitanicus*. Map depicting the distribution range of the Iberian endemic *Misolampus lusitanicus* (red spot). Purple dots correspond to the species records, including both recent and old, as well as previously published data **B** potential geographic distribution of *Misolampus lusitanicus*: Red indicates areas of high suitability, and blue, areas of low suitability. Species distribution model was generated using MaxEnt v 3.4.1 (Elith et al., 2006) and the set of WorldClim v 2.0 (Fick and Hijmans 2017) environmental variables.

Endemism of northern Portugal and northwestern Spain (Löbl et al. 2008) (Fig. 6). Published records are very scarce but distributed in the district of Braga (Portugal) and provinces of León, Ourense, Pontevedra, and Zamora (Spain) (Brême 1842; von Heyden 1870; Paulino de Oliveira 1894; Reitter 1917; De la Fuente 1934–1935; Español 1949, 1955, 1956; Español and Comas 1981; Novoa et al. 2014).

The material we studied includes recent representation from the provinces of León and Ourense in Spain, and from the Porto district in Portugal. To date, the species is only known from ten localities (Fig. 6A). The potential distribution map locates high suitable areas for this species mainly in the northwestern region of the Iberian Peninsula (Fig. 6B).

##### Notes on natural history.

*Misolampus lusitanicus* is a medium altitude species (altitudinal range 572–1680 m a.s.l.; 59% of records above 1000 m), typical of mountainous reliefs of northwestern Iberian Peninsula (Macizo Galaico-Leonés mountain range: Serra San Mamede-Queixa, Serra do Eixe, Serra do Gêres, Serra Segundeira y do Porto, Serra dos Ancares, Serras Occidentais and Montes de León). Geological substrates in its geographic range are mainly granite, gneiss and, to a lesser extent, quartzite, which form acid soils (Vera 2004; Oliveira and Quesada 2019a, b). It occupies meso- and supra-Temperate thermoclimatic belts, and more locally meso- and supra-Mediterranean, mostly in the Atlantic European biogeographic province, in high rainfall regions, with ombrotypes humid and hyperhumid (Rivas-Martínez 1987; Rivas-Martínez et al. 2002; Rivas-Martínez 2007). The species inhabits humid forest habitats, mainly of deciduous oak trees (*Quercus robur* L., *Q. pyrenaica*), hazel (*Corylus avellana* L.), birch (Betula pubescens ssp. celtiberica Rothm. & Vasc.), chestnut trees (*Castanea sativa*), and yews (*Taxus baccata* L.), but also heathlands and rocky open areas covered by broom shrubs (*Cytisus oromediterraneus* Rivas Mart. & al. and *C. scoparius*) (see Izco 1987; Rivas-Martínez 1987; Costa Tenorio et al. 2005) (Fig. 5E, F).

Adults are usually found at the base of trees, under bark, under stones or in leaf litter of forests (Español 1956; Español and Comas 1981), but also under stones in mountain shrub-lands (pers. obs.). It has also been found in densely reforested areas with *P. pinaster*, and also in chestnut groves (*C. sativa*). It has not been recorded in sympatry with any other species of *Misolampus*, but it has been found in company of *Coelometopus clypeatus* (Germar, 1813) (Tenebrionidae, Cnodalonini) (Español and Comas 1981). According to the limited available data, adults seem to be present all year round.

#### Misolampus ramburii

Taxon classificationAnimaliaColeopteraTenebrionidae

Brême, 1842

8DD087C6-088C-5B33-B1CE-C0C0E1F7FC35

Misolampus ramburii Brême, 1842: 82. Terra typica: “De l’Espagne meridionale”. Some authors wrote the species name with a single final -i (Rosenhauer 1856; Paulino de Oliveira 1894; Reitter 1917; De la Fuente 1934–1935; Palau 1945; Español 1949).

##### Studied material.

Spain – Andalucía: Almería: Fondón: 2 exx.; Sierra Bacares: 1900 (Escalera leg.): 3 exx.; Sierra Alhamilla, Almería, 1240 m, 36°59'25"N, 02°20'13"W, 30-XII-2003 (P. Barranco leg.): 1 ex.; Sierra de Gádor, 892 m, 36°55'32.18"N, 2°35'57.07"W, 27-III-2012: 3 exx.; Granada: Jayena, 30-VII-1920: 4 exx.; Puerto de la Mora, 1294 m, 37°15'19.71"N, 3°29'01.80"W, 26-III-2012: 2 exx.; Pista La Alcaicería-El Robledal (encinar), Sierra Tejeda, 1020 m, 36°57'07"N, 4°00'56"W, 5-I-2005: 1 ex.; Málaga: Málaga (Aragoncillo leg.) (Col. del Sr. Pérez Arcas): 1 exx.; Arroyo Güi, Torrox, 155 m, 36°46'36"N, 3°59'29"W, 23-XII-2000: 4 exx.; Lagos, Velez-Málaga, 102 m, 36°45'00"N, 4°00'28"W, 15-IV-2006: 2 exx.; Área El Pinarillo, Nerja, Sierra de Almijara, 485 m, 36°47'53"N, 3°50'55"W, 3-I-2003: 6 exx.; Área El Pinarillo, Nerja, Sierra de Almijara, 471 m, 36°47'52"N, 3°50'58"W, 4-I-2012: 3 exx.; Cerro El Cañuelo, Acantilados de Maro-Cerro Gordo, Nerja, 130 m, 36°44'57"N, 3°47'12"W, 29-XII-2007: 1 ex.; Carril Cuevas de Nerja-El Pinarillo, Sierra de Almijara, 340 m, 36°46'58"N, 3°50'24"W, 30-III-2018: 2 exx.; Alrededores Cuevas de Nerja, Maro, Nerja, 171 m, 36°45'46"N, 3°50'43"W, 2-XI-2018: 1 ex. – Murcia: Sierra Espuña: VIII-1943 (G. Menor leg.): 1 exx.

##### Diagnosis.

**Figure 7.**
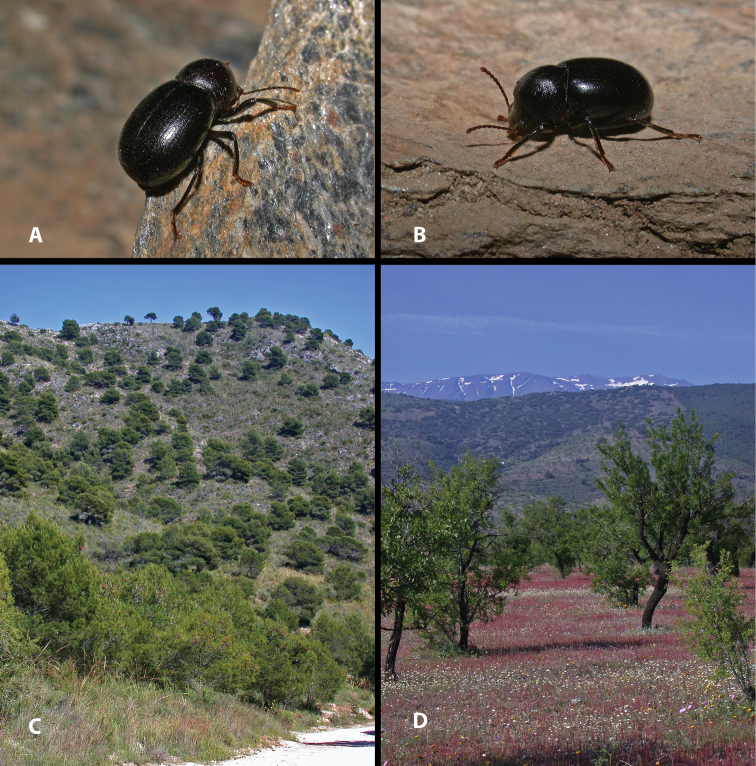
Live specimens and habitat of *Misolampus ramburii***A, B** adult *Misolampus ramburii* from Spain (**A** Sierra de Gádor, Almería **B** Sierra de Huétor, Granada); the specimens selected represent the diversity of sculptural patterns in elytra and pronotum, smoother in western populations, without elytral striae (**B**), marked in eastern areas (**A**) **C, D** two examples of typical habitat of *M. ramburii* from **C** coastal ravines with scattered *Pinus halepensis* (Maro, Málaga) and **D** slope of Sierra Nevada with open forests of *Q. ilex* and almond trees (Almería). Photographs by MGP.

Total length 9–11 mm (Reitter 1917; Español 1949). *Misolampus ramburii* is characterised by its shiny appearance and by presenting the anterior angles of prothorax slightly protruding forward. Pronotal punctation deep and dense, not as strong as in *M. gibbulus*, without granular areas. Elytra with shallow striae formed by series of punctures in longitudinal series, sometimes almost absent (Fig. 7A, B). Elytral inter-striae smooth. Female genitalia figured by Palmer (1998) and aedeagus by Español (1949). Adults present a marked variability in elytral sculpturing, smoother with elytral striae almost erased in the western populations of Sierra de Almijara and Sierra de Huétor (Fig. 7B); more marked in the eastern areas (Fig. 7A) (see taxonomic discussion).

##### Geographic distribution.

**Figure 8.**
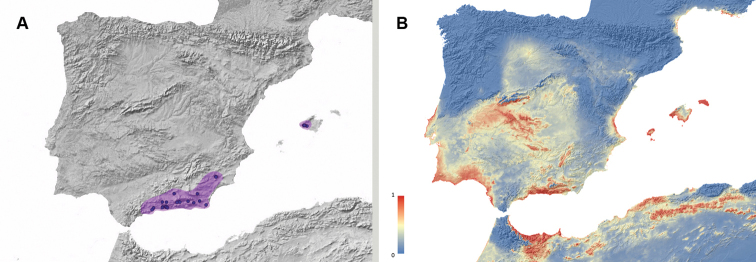
**A** Geographic distribution of *Misolampus ramburii*. Map of the Iberian Peninsula depicting the geographic distribution of *Misolampus ramburii* (purple spot), an endemic species to Spain. Blue dots correspond to the species records, including both recent and old, as well as previously published data **B** potential geographic distribution of *Misolampus ramburii*: Red indicates areas of high suitability, and blue, areas of low suitability. Species distribution model was generated using MaxEnt v 3.4.1 (Elith et al. 2006) and the set of WorldClim v 2.0 (Fick and Hijmans 2017) environmental variables.

Endemism of southeastern Spain and Mallorca in the Balearic Islands (Fig. 8). Records from Portugal, as Serra de Monchique (Paulino de Oliveira 1894; Reitter 1917; Löbl et al. 2008), are based on misidentifications (see taxonomic discussion). Published records are scarce, but covering most of the known species range, from the provinces of Almería, Granada, Málaga, Murcia, and the island of Mallorca (Rosenhauer 1856; von Heyden 1884 sub *M. scabricollis*; Reitter 1917; De la Fuente 1934–1935; Palau 1945; Cobos 1949; Español 1949, 1954a, 1954b, 1963; Sánchez-Piñero et al. 2013; Valladares et al. 2013). All published records are relatively old, except those from Almería and Granada. According to the current records, *M. ramburii* is restricted to the Betic Mountain ranges (Montes de Málaga, Sierra Nevada, Sierra de Huétor, Sierras de Tejeda and Almijara, Sierra de Filabres, Sierra de Gádor, Sierra Alhamilla, Sierra Espuña), and in Malllorca to the southwestern foothills of the Sierra de Tramuntana, including Palma Bay (Fig. 8A). The record of an unidentified *Misolampus* from Sierra de Contraviesa (Granada) (Español 1963), found in the company of *Coelometopus cobosi* Español, 1963, probably corresponds to *M. ramburii* (see taxonomic discussion).

Materials studied by us include specimens from all previously reported areas except Mallorca (not searched for). Records are recent for all localities except for those from the Murcia region (Sierra Espuña). The potential distribution map (Fig. 8B) shows that highly suitable areas are primarily located in the coasts and mountain ranges of the south of Almería, Granada, and Málaga and the northwest of Mallorca island, coinciding with the recorded presence of the species. The northwestern coast of the Iberian Peninsula and the mountain ranges of Northwestern Africa are also pointed as areas of high suitability.

##### Notes on natural history.

*Misolampus ramburii* is a low-medium mountain species, even sub-coastal, with an altitudinal range between 14 and 1673 m a.s.l. (60% of records below 1000 m of altitude); in Mallorca it is also found at low altitude, 14–398 m a.s.l. Lithological substrates of its area of occupancy are very diverse, due to the high geostructural complexity of the Betic Mountain ranges, but are mainly dolomites, limestones, slates, phyllites, mycaschists, and, very locally, plutonic rocks (Sanz de Galdeano 1997; Vera 2004; Oliveira and Quesada 2019a, 2019b). It inhabits usually the thermo- and meso-Mediterranean bioclimatic levels, and very locally at the supra-Mediterranean, with ombrotypes from semiarid to subhumid (Rivas-Martínez 1987; Rivas-Martínez et al. 2002; Valle 2003; Valle et al. 2004). According to the known localities and our observations, the species occurs in an extensive variety of habitats, with preference for more or less open forested areas with pines (*Pinus halepensis*, *P. pinaster*, *P. nigra*, both natural and reforested), oaks (*Quercus ilex*, *Q. faginea* and *Q. suber* in Sierra de Contraviesa), wild olive trees (Olea europaea var. sylvestris), and carob trees (*Ceratonia siliqua* L.), but also in shrub-lands of *Quercus coccifera* L., *Genista umbellata* (L’Hér.) Dum. Cours., *Rosmarinus officinalis* L., *Pistacia lentiscus* L., *Pistacia therebinthus* L., *Buxus balearica* Lam., *Maytenus europaeus* (Boiss.) Rivas Mart., *R. sphaerocarpa*, and *Cistus* and *Lavandula* L. species, among other typical shrubs (Rivas-Martínez and Costa 1987; Bolòs 1997; Valle 2003; Costa Tenorio et al. 2005). Sometimes, it has been found in almond tree crops (*Prunus dulcis* (Mill.) D.A. Webb) with scattered oaks (*Q. ilex*) (pers. obs.) (Fig. 7C, D).

Commonly found under bark or inside dead logs and stumps of pines (*P. halepensis*, *P. pinaster* and *P. nigra*), and oaks (*Q. ilex*), or under stones in forests and shrub-lands. Occasionally found under the lose bark of standing live isolated *Eucalyptus* trees. In the island of Mallorca, it has been found in oak forests (*Q. ilex*), under bark or under stones and leaf litter (Español 1949, 1954a). Adults can be found in autumn, winter, and spring, with no records in the summer months of August and September.

#### Misolampus scabricollis

Taxon classificationAnimaliaColeopteraTenebrionidae

Graells, 1849

C934E2FF-5E14-51F9-9F9C-20D7DEBDADA5

Misolampus scabricollis Graells, 1849: 621. Terra typica: “Guadarrama”. Graells (1851a) mentioned the unavailable name “Misolampus graellsi Dufour”.

##### Studied material.

Portugal – Guarda: 2 km al O de Vale de Estrela, 977 m, 40°29'35.7"N, 7°19'12.1"W, 18-IV-2011: 1 ex.; [6 km al SO de] Guarda, 11-X-1992: 7 exx.; S. de Estrella [Serra da Estrela] (Sanz leg.): 1 ex. – Portalegre: Monte Palheiros, 632 m, 39°20'03.91"N, 7°25'38.63"W, 10-III-2012: 12 exx.; Ribeira de Nisa, [4 km al NE de Nisa], 23-X-1990: 1 ex. Spain – Andalucía: Huelva: Andalucía: Huelva: Cortegana, 706 m, 37°53'55.6"N, 06°50'16.3"W, 3-I-2013: 2 exx.; Cortegana: Puerto del Corzo (hacia Gil Márquez), 664 m, 37°52'56.1"N, 06°50'42.3"W, 3-I-2013: 9 exx. – Castilla – La Mancha: Ciudad Real: Fuencaliente (Sierra Morena) (J. Cabré leg.): 1 ex.; Navas de Estena: 2 exx.; Puebla de Don Rodrigo: El Vivero, 39°02'29.2"N, 4°33'40.9"W, 5-III-2012: 1 ex.; Saceruela: 1 ex., plus 1 without label; Guadalajara: 3 km al O de La Mierla, 1023 m, 40°56'27.6"N, 3°16'13.4"W, 26-X-2013: 12 exx.; Alpedrete de la Sierra, hacia el Atazar, 17-IV-1992: 3 exx.; Retiendas: Embalse del Vado, 931 m, 41°00'09.8"N, 3°17'41.6"W, 26-X-2013: 2 exx.; Umbralejo, 1256 m, 41°07'33.2"N, 3°10'39.4"W, 26-X-2013: 18 exx.; Toledo: Belvís de la Jara (N502 km 153), 584 m, 39°43'59.3"N, 4°58'14.6"W, 1-XI-2008: 1 ex.; Las Honfrías, Robledo del Mazo, 39°35'48"N, 04°53'15"W, 9-II-2011: 1 ex.; Navamorcuende: Sierra de San Vicente: El Piélago, 1154 m, 40°08'34.4"N, 4°44'09.2"W, 27-XII-2011: 3 exx.; Sierra de San Vicente: El Piélago, 1224 m, 40°08'03.91"N, 4°43'48.79"W, 13-V-2012: 1 ex. – Castilla y León: Ávila: Chamartín de la Sierra: Castro de la Mesa de Miranda, 40°43'24.7"N, 4°56'57.6"W, 10-II-2013: 1 ex.; [Navarredonda de] Gredos: 1 ex.; [Navarredonda de] Gredos (J. Ardois leg.): 10 exx.; Arenas [de San Pedro] (J. Ardois leg.): 1 ex.; Ávila (197) (Pérez leg.): 1 ex.; Casillas, 1158 m, 40°19'25.6"N, 4°35'14.0"W, 16-XI-2012: 1 ex.; Casillas: 4 exx.; Las Navas [del Marqués]: [Sierra de] Guadarrama (G. Schramm leg.): 75 exx.; 5 km S Navas del Marqués, 40°33'24.4"N, 4°19'32.5"W, 1-IV-2013: 1 ex.; Mombeltrán – Navalperal: 2 exx.; Navalperal [de Tormes], VII-1904 (Escalera leg.): 1 ex.; Navamorcuende (Ardois leg.): 1 ex.; Navas del Marqués: Carretera de Valdemaqueda, 1021 m, 0°32'14.3"N, 4°20'26.5"W, 20-III-2010: 1 ex.; Peguerinos: Valle de Enmedio, 1-VII-1992: 2 exx.; Puerto de Casillas, 1590 m, 40°20'37.1"N, 4°35'06.7"W, 15-V-2011: 1 ex.; Sierra de Gredos: 2 exx.; Valle de Iruelas, 10-V-1919 (J. Abajo leg.): 8 exx.; Valle de Iruelas, V-1920: 7 exx.; Villarejo [del Valle]): 1 ex. plus 1 without label; Burgos: Quemada, 848 m, 41°43'20.4"N, 3°33'00.3"W, 9-V-2013: 4 exx.; Salamanca: 1 km al N del Puerto de Perales, 884 m, 40°15'18.3"N, 6°41'22.2"W, 16-IV-2011: 2 exx.; Navasfrías, 959 m, 40°17'03.1"N, 6°49'49.1"W, 23-XII-2011: 9 exx.; Peña de Francia: 1 ex.; Puerto de Perales, 917 m, 40°14'46.2"N, 6°41'20.5"W, 22-XII-2011: 3 exx.; Serradilla del Llano, 13-X-1992: 2 exx.; Segovia: Balsaín (C. Bolívar leg.): 1 ex.; Balsaín (J. Abajo leg.): 1 ex.; Balsaín (J. Ardois leg.): 15 exx.; Collado Ventoso, 1964 m, 40°47'13.2"N, 4°02'32.8"W, 11-VIII-2013: 3 exx.; El Espinar: 1 exx.; Puerto de Los Cotos – Dos Hermanas, 40°49'27.1"N, 3°57'51.4"W, 19-XI-2012: 1 ex.; Puerto de Los Cotos – Dos Hermanas, 1900 m, 2-IX-1991: 2 exx.; Puerto de Navacerrada, 40°47'11"N, 4°01'05"W, 13-V-2012: 1 ex.; Puerto de Navacerrada, 40°47'17.83"N, 4°00'36.27"W, 30-V-2012: 1 ex.; Zamora: Santa Ana, 872 m, 41°42'17.66"N, 6°24'22.65"W, 25-IV-2012: 11 exx. – Extremadura: Cáceres: Alcuéscar, I-1894: 2 exx.; Carretera Villamiel – San Martín de Trevejo, 868 m, 40°11'43.8"N, 6°47'30.3"W, 23-XII-2011: 6 exx.; Casares de Las Hurdes: Puerto de Robledo, 1074 m, 40°27'07.06"N, 6°17'48.82"W, 17-IV-2012: 4 exx.; Hurdes: 1 exx.; Madrigal [de la Vera]: 1 exx.; Madrigal [de la Vera] (J. Ardois leg.): 21 exx.; Pico Villuercas, 1394 m, 39°28'19.72"N, 5°23'54.70"W, 12-V-2012: 7 exx. – Madrid: Dehesa de Braojos, 1400 m, 41°03'27.4"N, 3°38'51.1"W, 12-X-2013: 1 ex.; Cadalso [de los Vidrios] (J. Ardois leg.): 3 exx.; Cercedilla, 1460 m, VII-1945 (L. Esteban leg.): 1 ex.; Cercedilla, 1500 m, VIII-1935 (J. Hernández leg.): 4 exx.; Cercedilla, [Sierra de] Guadarrama (G. Schramm leg.): 11 exx.; Cercedilla, [Sierra de] Guadarrama (E. Zarco leg.): 2 exx.; Cercedilla (Lauffer leg.): 1 exx.; Cercedilla (Moróder leg.): 10 exx.; Cercedilla (Exp. del Museo): 20 exx.; Cercedilla (C. Bolívar leg.): 23 exx.; Cercedilla, 25-VII-1926: 1 ex.; Cercedilla (J. Ardois): 5 exx.; Cercedilla (Museo): 5 exx.; Cercedilla: 6 exx.; Cercedilla: El Ventorrillo, 1480 m: VIII-1960 (J. Abajo leg.): 2 exx.; Cercedilla: El Ventorrillo, 1478 m, 40°45'17.3"N, 4°01'21.6"W, 11-VI-2013: 11 exx.; Cercedilla: Estación Alpina, 1460 m (J. Abajo leg.): 1 ex.; Cercedilla: Estación Alpina, 1500 m: 2 exx.; Cercedilla: Estación Alpina, XII-1941 (E. Zarco leg.): 1 ex.; El Escorial (J. Dusmet leg.): 1 ex.; El Escorial (*Misolampus scabricollis* Graells): 1 ex. plus 1 without label; El Escorial, 10-V-1926: 3 exx.; El Escorial, 10-VI-1927: 2 exx.; El Escorial, 20-V-1925: 2 exx.; El Escorial, 22-V-1953 (W. Steiner leg.) (T-29) (*Misolampus scabricollis* Graells, F. Español det.): 4 exx.; El Escorial (C. Bolívar leg.): 4 exx.; El Escorial: 4 exx.; El Escorial (Lauffer leg.): 5 exx.; El Escorial: Cuelgamuros, 1337 m, 40°38'53.8"N, 4°09'19.8"W, 10-VI-2013: 9 exx.; El Escorial: Puerto [de Malagón]: 1 ex.; El Paular (Exp. del Museo): 11 exx.; Garganta de Los Montes, 1346 m, 40°54'46.9"N, 3°40'05.5"W, 26-V-2013: 2 exx.; Lozoyuela, 1288 m, 40°55'31.4"N, 3°39'44.9"W, 26-V-2013: 5 exx.; Manzanares [El Real], 30-III-1928: 1 exx.; Manzanares El Real, 1156 m, 40°45'28.1"N, 3°54'56.0"W, 28-II-2012: 3 exx.; Pelayos de la Presa, 799 m, 40°20'19.40"N, 4°21'34.84"W, 3-III-2001: 1 ex.; Puerto de Cotos, 12-VIII-1925: 1 ex.; Puerto de La Hiruela, 1354 m, 41°03'42.5"N, 3°28'36.8"W, 6-IV-2011: 1 ex.; Puerto de La Puebla, 1633 m, 41°02'27.7"N, 3°28'48.9"W, 27-IV-2011: 3 exx.; Puerto de Navacerrada, 18-VIII-1923): 1 ex.; Puerto de Navacerrada (E. Zarco leg.): 2 exx.; San Lorenzo del Escorial, 40°35'58"N, 4°09'42"W, 14-III-2015 (A. Sánchez Vialas): 2 exx.; Santa María de la Alameda (estación), 1-IV-1991: 1 ex.; Santa María de la Alameda, 1437 m, 40°36'11.11"N, 4°15'18.93"W, 30-V-2012: 1 ex.; Sierra de Guadarrama (Lauffer leg.): 2 exx.; Tablada, 12-V-1957 (J. Álvarez leg.): 1 ex.; Valdemanco, 1090 m, 40°51'10.5"N, 3°38'48.5"W, 8-V-2013: 1 ex.

##### Diagnosis.

**Figure 9.**
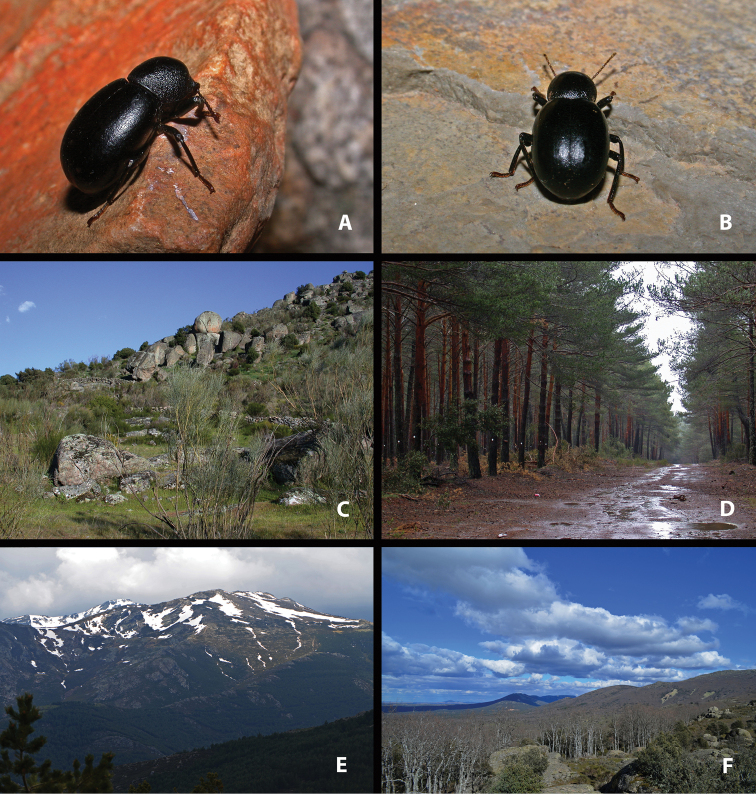
Live specimens and habitat of *Misolampus scabricollis***A, B** live adult specimens of *Misolampus scabricollis* from Spain (**A** Sierra de Guadalupe, Cáceres **B** Las Honfrías, Montes de Toledo) **C–F** typical habitats of *M. scabricollis* (**C** granitic outcrops with *Cytisus*, *Juniperus communis*, and *Quercus ilex* along the Sistema Central Mountain Chain, Avila **D** densely reforested area with *Pinus sylvestris* at Santa Ana, Zamora) **E***Pinus sylvestris* forests at the southern slopes of Pico del Lobo, Guadalajara **F** dense forests of *Quercus pyrenaica* at Montes de Toledo). Photographs by MGP.

Total length 11–13 mm (Graells 1849, 1851a, 1851b; Reitter 1917; Español 1949; López-Pérez 2014a). Pronotum with strong punctation intermixed with raised granules and small tubercles, particularly developed on the lateral sides, which gave them a strongly rugose appearance. Propleural sides with dense strong punctation. Elytra smooth, without traces of striae, series of punctures, or tubercles (Fig. 9A, B). Female genitalia figured by Palmer (1998) and aedeagus by Español (1949). Morphological variability seems to be restricted to individual variation in size and in the extent of the pronotal rugose areas.

##### Geographic distribution.

**Figure 10.**
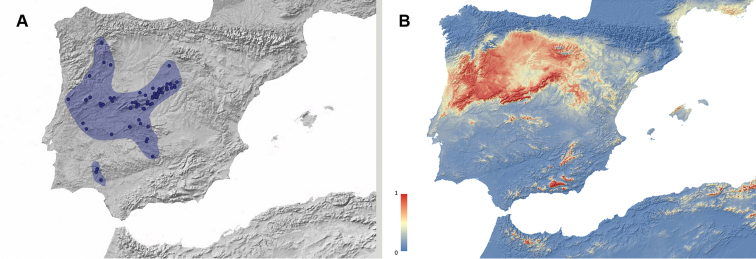
**A** Geographic distribution of *Misolampus scabricollis*. Distribution range of the Iberian endemic *Misolampus scabricollis* (dark blue spot). Blue dots correspond to the species records, including both recent and old, as well as previously published data. The populations from Huelva (southwestern Spain) remain isolated, since no intermediate populations are known in a distance of at least 150 km, however intervening habitat seems favourable in many areas **B** potential geographic distribution of *Misolampus scabricollis*: Red indicates areas of high suitability, and blue, areas of low suitability. Species distribution model was generated using MaxEnt v 3.4.1 (Elith et al. 2006) and the set of WorldClim v 2.0 (Fick and Hijmans 2017) environmental variables.

Endemism of Portugal and Spain (Löbl et al. 2008) (Fig. 10). Bibliographic records are scarce, covering a large portion of the centre and western areas of the Iberian Peninsula, including Aveiro, Bragança, Alto Douro, and Guarda in Portugal, and the provinces of Ávila, Cáceres, Huelva, Madrid, Ourense, and Segovia in Spain (Graells 1849, 1851a, 1851b; Seidlitz 1867; Paulino de Oliveira 1894; Reitter 1917; De la Fuente 1934–1935; Español 1949; López-Pérez 2014a; Novoa et al. 2014). Published records of *M. scabricollis* from Sierra Espuña (von Heyden 1884), Murcia (De la Fuente 1934–1935) and Sierra de Alcaraz (Reitter 1917) are erroneous and probably correspond to *M. subglaber*. *Misolampus scabricollis* is widely distributed throughout the main mountain ranges of the central and western areas of the Iberian Peninsula (Sistema Central, Sierra de Gata, Sierra de Guadalupe, Montes de León, Montes de Toledo, eastern Sierra Morena, Serra da Estrêla), with an apparently isolated population in the western extreme of Sierra Morena (Sierra de Aracena, province of Huelva) separated ca. 240 km from the eastern population of this same mountain system (Fig. 10A).

All previously existing records except those of Huelva and Ourense, correspond to data published more than 70 years ago. The material studied or collected by us, includes records from all provinces of Spain previously reported in the literature, except from Ourense, with the addition of new records from Castelo Branco and Portalegre in Portugal, and from the provinces of Burgos, Ciudad Real, Guadalajara, Salamanca, Toledo, and Zamora in Spain. All these new records correspond to recent observations, together with old ones for Ciudad Real and Salamanca. The potential distribution map for this species (Fig. 10B) locates the main high suitable areas in central and western regions and along mountain ranges of the Iberian Peninsula. The SDM does not consider the isolated population of Sierra de Aracena as present in a high suitability area.

##### Notes on natural history.

*Misolampus scabricollis* is a medium-low mountain species, with an elevation range of 224 to 1964 m a.s.l. (78% of the records are above 800 m, 56 % above 1000 m of altitude). Lithological materials of its area of occupancy are siliceous and very diverse, mainly granites, schists, gneisses, quartzites, and plutonic rocks (Vera 2004; Oliveira and Quesada 2019a, 2019b). Most of its distribution area is located in the meso- and supra-Mediterranean thermoclimatic belts, and more locally at higher altitude in the oro-Submediterranean (exceptionally, one location is in the upper thermo-Mediterranean: Niebla, province of Huelva), with ombrotypes dry, subhumid and humid (Rivas-Martínez 1987; Rivas-Martínez et al. 1987, 2002; Valle et al. 2004; Rivas-Martínez 2007). It occurs in a large variety of forested habitats with different degrees of coverage, usually composed by pines (*Pinus sylvestris*, *P. pinea*, *P. pinaster*, natural or reforested), oaks (perennial: *Quercus suber*, *Q. ilex*; deciduous: *Q. pyrenaica*, *Q. faginea*) and chestnut trees (*Castanea sativa*), as well as substitution shrubs, mainly of *Cistus*, *Cytisus*, *Ulex* L. and *Genista* L. (Ladero 1987; Rivas-Martínez 1987; Rivas-Martínez et al. 1987; Valle 2003; Costa Tenorio 2005) (Fig. 9C–F).

According to our observations, *M. scabricollis* is usually found inside dead and decaying tree trunks, or under bark, usually in standing or lying pine logs, oaks, and chestnut trees. These observations are coincident with the few disperse available data on the habitat of this species (Graells 1851a, 1851b; López-Pérez 2014a). Areas covered by dense bushes of *Q. pyrenaica* and *Q. ilex* (recovering after fires or logging) are also frequently used by this species. *Misolampus scabricollis* can also be found in areas reforested with pines, and under stones, small pieces of wood, or inside tight clusters of branches, in shrub areas dominated by *Cytisus scoparius*, *C. oromediterraneus*, and less frequently by *Cistus ladanifer* (Fig. 9C–F). They are usually more easily found on logs and under stones at the edge of dense forests, but they can also be found deep inside the forest or in nearby grasslands.

This species is usually found forming small groups of 2–21 specimens in a single log. Graells (1851a, 1851b) reported groups of five or six specimens per log in the Guadarrama Mountains. According to Graells (1851a, 1851b), when disturbed they pretend to be dead (thanatosis) and expel an unpleasant light odour.

*Misolampus scabricollis* has been found in microsympatry with *M. gibbulus* along western Sierra Morena (Huelva), northern Extremadura (Cáceres), Montes de Toledo (Toledo) and southern slopes of the Sistema Central (Madrid, Ávila, Toledo) (Fig. 1E), however, *M. scabricollis* is usually found at higher altitudes than *M. gibbulus*. Adults can be found across most of the year, but are more easily encountered during the wetter, colder, months (October to May). It is often found in company of *Coelometopus clypeatus* in old chestnut trunks.

#### Misolampus subglaber

Taxon classificationAnimaliaColeopteraTenebrionidae

Rosenhauer, 1856

5BA4FFAA-A497-54A9-AFFD-0FED30083839

Misolampus subglaber Rosenhauer, 1856: 204. Terra typica: “in der Sierra de Ronda”.

##### Studied material.

Spain – Andalucía: Córdoba: Córdoba: 1 ex.; Granada: Güejar Sierra: 1 ex.; La Sagra (Escalera 1900): 4 exx.; Puebla de Don Fadrique (Escalera 1900): 5 exx.; Puebla de Don Fadrique: Nablanca, 1517 m, 38°00'23.6"N, 2°28'28.2"W, 10-IV-2011: 2 exx.; Valdeiglesias, 975 m, 36°56'49.3"N, 4°04'29.6"W, 24-X-2019: 3 exx.; Jaén: 3 km SO Aldeaquemada, 38°23'53.7"N, 3°24'00.5"W, 7-III-2012: 5 exx.; [3 km al SO de] Aldeaquemada, 26-IV-1992: 2 exx.; Cazorla: 7 exx.; Vadillo de Castril, Sierra de Cazorla, 995 m, 37°55'14"N, 2°55'50"W, 8-V-2008 (D. Ruiz leg.): 1 ex.; Santa Elena, carretera hacia La Aliseda, 768 m, 38°20'18.0"N, 3°32'56.8"W, 11-IV-2011: 3 exx.; Santa Elena, carretera hacia La Aliseda, 795 m, 38°20'53.1"N, 03°33'20.6"W, 28-XII-2010: 1 ex.; Santiago de la Espada (J. Martínez): 1 ex.; Segura [de la Sierra]: 1 ex.; Sierra Morena (Laguna leg.): 1 ex.; Málaga: 3 km al E de Jubrique, 786 m, 36°33'37.5"N, 5°10'40.9"W, 14-IV-2013: 6 exx.; Nerja: 1 ex.; El Colmenar, Gaucín, P.N. Los Alcornocales, 255 m, 36°32'29"N, 5°23'22"W, 17-II-2018 (S. Yubero leg.): 3 exx.; Carril Llanada de Sedella-Bco. de Valdeinfierno, Sierras de Tejeda y Almijara, 1495 m, 36°53'15"N, 3°56'40"W, 4-I-2017: 2 exx. – Castilla – La Mancha: Albacete: Agramón: 2 exx., plus 1 without label; Alcaraz: 3 exx.; Calar del Mundo, V-1904 (G. Schramm leg.): 1 ex.; Cañadillas, 15-VI-1938: 1 ex.; Cañadillas, 16-VI-1938: 1 ex.; Cañadillas, 17-VII-1938: 1 ex.; Los Collados, 20-II-1938: 1 ex.; Molinicos: 1 ex., plus 4 exx. without labels; Riópar, 25-VII-1926: 1 ex.; San Juan de Alcaraz [Fábricas de Riópar] (Paz leg.): 1 ex.; Ciudad Real: Solana del Pino: Puerto Madrona, 38°25'07.3"N, 4°03'33.1"W, 06-III-2012: 3 exx.; Cuenca: Puerto de Cabrejas, 1167 m, 40°04'17.9"N, 2°18'39.5"W, 10-XI-2012: 1 ex. – Murcia: Jumilla: 3 exx.

##### Diagnosis.

**Figure 11.**
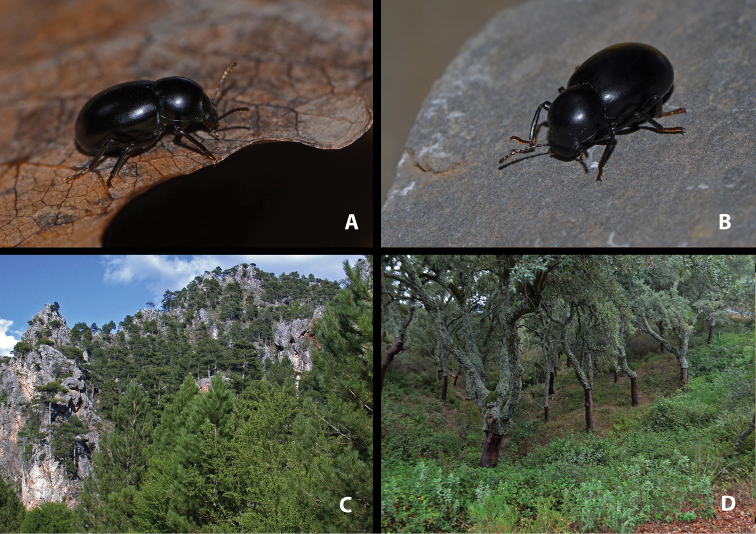
Live specimens and habitat of *Misolampus subglaber***A, B** live specimens of *Misolampus subglaber* from Spain (**A** Valdeiglesias, Sierra Tejeda, Granada **B** Miranda del Rey, Sierra Morena, Jaén) **C, D** general habitat occupied by *M. subglaber* (**C** limestone outcrops with *Pinus nigra* along the Sierra de Alcaraz, Albacete **D***Quercus suber* forests at Cortes de la Frontera, Sierra de Grazalema, Málaga). Photographs by MGP.

Total length 10–12 mm (Reitter 1917; Español 1949). Species clearly characterised by the combination of the following traits: smooth silky appearance; antennae graceful, reaching the base of pronotum; pronotal punctation very fine and sparse on the disc, somewhat stronger and denser to the sides; elytral punctation very fine and irregular, not forming longitudinal series of points or striae (Reitter 1917; Español 1949; Palmer 1998) (Fig. 11A, B). Female genitalia figured by Palmer (1998). The species has been studied karyologically and presents 2n = 20 chromosomes (Palmer and Petitpierre 1997). Morphological variability within this species seems limited to the depth and density of pronotal punctation, and it does not appear geographically structured.

##### Geographic distribution.

**Figure 12.**
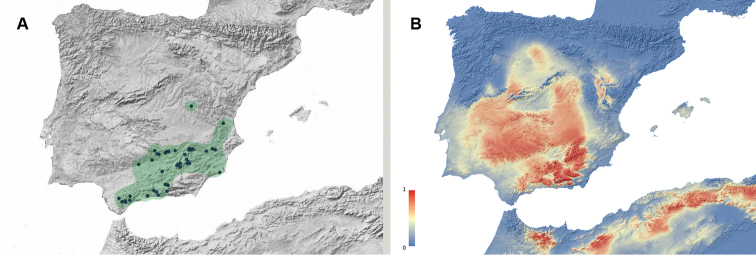
**A** Geographic distribution of *Misolampus subglaber*. Distribution range of *Misolampus subglaber* (green spot). Blue dots correspond to the species records, including both recent and old, as well as previously published data. Cuenca population is isolated from all other known populations by a distance of 150 km. The old bibliographic record from Cartagena (province of Murcia, south western Spain) requires confirmation **B** potential geographic distribution of *Misolampus subglaber*: Red indicates high suitable areas, and blue, areas of low suitability. Species distribution model was generated using MaxEnt v 3.4.1 (Elith et al. 2006) and the set of WorldClim v 2.0 (Fick and Hijmans 2017) environmental variables.

Endemism of southeastern Spain (Löbl et al. 2008) (Fig. 12). Published records are scarce, but well distributed throughout Andalucía: Granada, Jaén, Málaga; Castilla – La Mancha: Albacete; Comunidad Valenciana: Valencia; and Murcia (Rosenhauer 1856; Piochard 1866; Reitter 1917; De la Fuente 1934–1935; Cobos 1949; Español 1949, 1960; Molino Olmedo 1996; Palmer and Petitpierre 1997; Ibáñez Orrico 2002; Pérez and López-Colón 2010; López-Pérez 2014a sub *M. erichsoni*, 2014b; Grimm and Aistleitner 2009; Bujalance de Miguel 2015). Records are distributed through time in all areas, except for the recent one from Valencia region (Fig. 12A).

The material studied or collected by us includes specimens from all provinces reported in the literature, except from Valencia. In addition, we studied material from the provinces of Córdoba, Ciudad Real and Cuenca; specimens of Ciudad Real and Cuenca are represented by recent collections (2012). According to these data, *M. subglaber* is located in the Betic Mountain range (Sierras del Campo de Gibraltar, Serranía de Ronda, Sierra Nevada, Sierras de Tejeda and Almijara, Sierra de Cazorla, Sierra de Alcaraz, Sierra de Cartagena), eastern and central Sierra Morena mountain range, and two apparently isolated populations in the Southern Iberian mountain range (Serranía de Cuenca and Sierra de Malacara, separated between them by ca. 150 km). There is a gap of records in the arid regions of the southeastern end of Spain, throughout the provinces of Almería and southern Murcia, including the eastern half of Sierra Nevada and Sierra de Filabres. The record from Cartagena, Murcia (Reitter 1917), requires further confirmation (Fig. 12A). The potential distribution map identifies the Betic Mountain ranges as the most suitable area for the species. The coastal areas of Almería, Granada, and Málaga provinces are however not included as very suitable. The southern Iberian Plateau and the northwestern African mountain ranges are also suggested as areas of high suitability for the species occurrence (Fig. 12B).

##### Notes on natural history.

*Misolampus subglaber* behaves as a low-medium altitude montane element, distributed within an altitudinal range of 56 to 1662 m a.s.l. (with 61% of its records above 800 m). Geological substrates along its distribution area are diverse, both acid and basic, including mainly sandstones, limestones, dolomites, slates, gneisses, schists and mycaschists (Sanz de Galdeano 1997; Vera 2004; Oliveira and Quesada 2019a, 2019b). *Misolampus subglaber* occupies mostly the thermo- and meso-Mediterranean thermoclimatic belts and locally supra-Mediterranean, in areas with ombrotype semiarid, dry, subhumid and, exceptionally, humid (Rivas-Martínez 1987; Rivas-Martínez et al. 2002; Valle 2003; Rivas-Martínez 2007). It occurs on an extensive variety of pre-forest and forest systems, more or less dense and open, including oaks (deciduous: *Quercus pyrenaica*, *Q. canariensis* and *Q. faginea*; perennial: *Q. suber* and *Q. ilex*) and pines (natural or reforested: *Pinus nigra*, *P. pinaster*, *P. halepensis*, and *P. sylvestris*), all of them usually with diverse undergrowth (Alcaraz Ariza and Peinado Lorca 1987; Peinado Lorca and Martínez Parras 1987; Laguna 1997; Valle 2003; Costa Tenorio et al. 2005) (Fig. 11C, D).

Adult specimens of *M. subglaber* have been found at the base and under mosses of old oak trunks and inside hollow branches on the ground (*Q. suber*, *Q. pyrenaica*, *Q. canariensis*, *Q. faginea*) (Fig. 11D), inside rotten logs, and under stones and leaf litter in pine forests (*P. nigra*, *P. pinaster*, *P. halepensis*) (Piochard 1866; Español 1960; Molino Olmedo 1996; Ibáñez Orrico 2002; pers. obs.). Pérez and López-Colón (2010) found a specimen inside a natural cavity in the province of Jaén, where it possibly came by stochastic passive dispersal. Often found in groups (Español 1960; pers. obs.).

Molino Olmedo (1996) found larvae inside decaying wood of branches and logs of *Q. pyrenaica*, *Q. canariensis*, *Q. faginea*, *Q. suber*, *Q. ilex* and *P. pinaster* and Ibañez Orrico (2002) on rotten logs of *P. halepensis* (however, the larva of *M. subglaber* has not been described yet). According to Molino Olmedo (1996), *M. subglaber* is a typical saproxylic species.

The general distribution area occupied by *M. subglaber* (Fig. 12) is largely coincident with that of *M. ramburii* (Fig. 8), however they have not been found in microsympatry, a possible indication of ecological segregation between them. Adults are mainly active in fall, winter and spring, but can be found all year round (pers. obs.). Large larvae and pupae have been observed at the end of August in Valencia (Ibañez Orrico 2002).

### Identification key for adult specimens of the genus *Misolampus* (modified from Español 1949)

**Table:** 

1	Elytra with series of deep to shallow punctures forming strongly to almost erased, excavated striae; additional series of punctures often present on the elytral intervals (Fig. 14A, B). Small size (6.9–12 mm)	**2**
–	Elytra without any trace of longitudinal series of punctures forming striae (Fig. 14C, D); if very weak striae are present, then interstriae show slightly raised longitudinal series of more or less developed and separated tubercles (Fig. 14D). Medium to small size (7.5–14 mm)	**3**
2	Anterior angles of pronotum slightly prominent (Figs 15A, 1C). Elytra with poorly marked striae, formed by longitudinal series of shallow to almost erased punctures; interstriae smoothly curved (Figs 7, 14A)	***M. ramburii***
–	Anterior angles of pronotum markedly prominent (Figs 15B, 16D). Elytra with strong to shallow striae, formed by longitudinal series of deep to shallow punctures; interstriae clearly convex (Figs 1, 14B)	***M. gibbulus***
3	Anterior angles of pronotum rounded, forming an open angle (Figs 16E, F). Elytra with shallow to almost erased striae, often showing longitudinal series of more or less developed elongated tubercles, or sometimes shallow fossae on the interstriae, better marked on the second half of the elytra and on the sides (Fig. 14D). Medium size (10–14 mm), long antennae (Fig. 3)	***M. goudotii***
–	Anterior angles of pronotum forming a widely acute to right angle (Figs 16A, B, 17). Elytra without any trace of striae or tubercles. Medium to small size (7.5–13 mm), relatively short antennae	**4**
4	Pronotum sculpture formed by deep large confluent punctures (Fig. 16A); the surface between punctures progressively transforms in irregular raised areas that become small irregular granules and wrinkles, giving a strongly rugose appearance to the pronotum sides (Figs 9, 16A). Medium size (11–13 mm)	***M. scabricollis***
–	Pronotum sculpture formed by deep to shallow, dense or sparse, never confluent, well-defined punctures which cover all the pronotal surface, including the lateral sides, which can present somewhat more confused punctation, but not forming rugose areas (Fig. 16B). Large to small size	**5**
5	Elytra covered by dense punctures somewhat confused or partially erased at the disc. Pronotum sculpture formed by deep, dense, well-defined punctation. Antennae relatively short, not reaching the base of pronotum (Fig. 5). Small size (7.5–8 mm)	***M. lusitanicus***
–	Elytra covered with very fine, shallow and sparse punctation that gave a silky shine to the elytral surface (Fig. 14C). Pronotum sculpture formed by shallow, spaced, and very fine well-defined punctation (Fig. 16B). Antennae usually reaching the base of pronotum (Fig. 11). Medium size (10–12 mm)	***M. subglaber***

## Discussion

### Comments on the taxonomy of Misolampus

North African *Misolampus* were described originally as three independent entities: *M. goudotii* from Tanger in northwestern Morocco (Guérin-Méneville 1834), *M. erichsoni* from Algeria (Erichson in Wagner 1841; Vauloger de Beaupré 1900; Reitter 1917) (Fig. 3A), and *M. peyerimhoffi* from the High Atlas in Morocco (Antoine 1926) (Fig. 3B). Additional variability was recorded, but not published, in the labels assigned by M. Martínez de la Escalera to specimens from the Rif region (“M. g. var. riffensis” Escalera *in litt*.; “M. g. var. laevior” Alluaud *in litt*.) and from Ifni (“M. g. var. ifnicus” Escalera *in litt*.) (Fig. 3C) at the MNCN collection (París García et al. 2011). Antoine (1949) described morphologically intermediate populations (Middle Atlas) and showed that male genital structures were similar between the three taxa. Accordingly, Antoine (1949), followed by Español (1949, 1954a), considered that the morphological traits used to separate the three described North African taxa were insufficient, and treated them as subspecies (*M. g. goudotii*, *M. g. erichsoni*, and *M. g. peyerimhoffi*). Español (1953, 1967) went further, and suggested that *M. g. erichsoni* should be included in the synonymy of *M. goudotii*, while Kocher (1958) indicated that all three taxa were just local varieties of a unique taxon. However, the criterion of Español (1953, 1967) and Kocher (1958) was not followed by subsequent authors (Löbl et al. 2008). Meanwhile, the morphological variability implied by Martínez de la Escalera and Vauloger identifications (in litt.), raises further problems for the characterisation of North African populations as subspecies.

Characters initially used for separation of the North African taxa were: pronotal punctation, shape of the anterior margin of the pronotum, shape and sculpture of the propleurae, and width of the second interstria on the elytra (Vauloger de Beaupré 1900; Antoine 1949; Español 1954a). A close examination of the specimens studied by Vauloger and Escalera (see materials and methods) reveals that some of the Rif specimens present intermediate traits between the specimens of the Tingitane Peninsula (Tanger, western Rif) and those from the Middle and High Atlas (Fig. 3B). At the same time, specimens from Ifni (Fig. 3C), roughly located at the coastal western end of the Anti-Atlas mountains, are more similar morphologically to the specimens from the Rif than to those geographically closer from the High Atlas.

Morphological similarity between specimens located in geographically isolated areas, separated by hundreds of kilometres, reflects that the morphological diversity documented across populations, lies within the phenotypic variability of a single evolutionary entity, rather than being a consequence of ancient isolation processes (Montori et al. 2008; Gonçalves et al. 2009). Alternatively, the observed morphological diversity could be consequence of a rapid response to recent geographic isolation of local populations subjected to local strong selective pressures (Velo-Antón et al. 2007). These hypotheses could be tested by genetic analyses, since the phylogeographic outcome of these two processes would be markedly different in each case: Geographically unstructured nuclear marker networks, accounting for the lack of geographic structure at the morphological level, with or without deep mtDNA lineage differentiation in the first case (Recuero and García-París 2011); or alternatively, geographically congruent nuclear and mtDNA marker phylogeographic patterns, with recent, shallow, multiple mtDNA lineage differentiation, accounting for the recency of the isolation processes, not enough to allow sorting out morphological differences, in the second case (Vörös et al. 2006; Rodríguez-Flores et al. 2017). However, none of these processes is consistent with the recognition of independent evolutionary units within North African *Misolampus*, and therefore we consider necessary to synonymise all three subspecies (*M. goudotii* Guérin-Méneville, 1834 = *M. erichsoni* Vauloger de Beaupré, 1900, syn. nov. = *M. peyerimhoffi* Antoine, 1926, syn. nov.), retaining thus a single North African species: *M. goudotii* Guérin-Méneville, 1834. The morphological similarity between the Balearic specimens and the Eastern Moroccan and Algerian ones drove Palmer and Cambefort (2000) to consider a very recent origin for the Balearic populations, possibly as a consequence of human-mediated dispersal.

There has been some confusion in the identification of specimens of *Misolampus* from southern Portugal (Serra de Monchique). Specimens from that region often present not strongly marked elytral striae, and relatively smooth thoracic impressions (Fig. 1A, B), resembling *M. ramburii* (Fig. 7A, B). However, a close examination of the Serra de Monchique specimens (Foia, Monchique, São Marcos da Serra) indicates that based on all other characters (mainly, prothorax morphology, and pronotal punctation), they correspond to *M. gibbulus*. The morphological differentiation shown by the population of *M. gibbulus* from Serra de Monchique with respect to other populations of the species, is quite marked, and led Reitter (1917), Paulino de Oliveira (1894) and De la Fuente (1934–1935) to mention erroneously the presence of *M. ramburii* in Serra de Monchique.

A similar situation occurs within *M. ramburii*. Specimens from populations of Granada (Sierras de Contraviesa and Huétor) have smoother pronotal sculpture, and less marked, almost absent elytral striae (Fig. 7B), while specimens from Almería show stronger sculpturing in elytra and pronotum, with elytral striae, marked by a series of aligned punctation, faint, but visible (Fig. 7A). This contrasting variation is probably the reason Español (1963) reported an unidentified species of *Misolampus* from the Sierra de Contraviesa. Lack of elytral striae made these specimens key to *M. subglaber*, *M. lusitanicus*, or *M. scabricollis* using Reitter’s (1917) identification table, but other characters, including pronotal structure, allow for an easy separation.

These evident patterns of morphological differentiation within *M. ramburii*, *M. gibbulus*, and *M. goudotii* may reflect a relatively recent history of isolation across populations, probably consequence of the existence of multiple isolated Pleistocene refugia (Abellán and Svenning 2014), as proposed for other flightless Iberian Coleoptera (Sánchez-Vialas et al. 2020). In any case, these hypotheses require phylogeographic analyses to be properly tested.

### Historical population continuity and current conservation status

Species of *Misolampus* have often been considered to present allopatric or, at most, parapatric distributions (Palmer 1998; Palmer and Cambefort 2000). However, old records of *Misolampus* are, in most species, scarce and unevenly distributed. Indeed, by filling large gaps where no records were present, the newly gathered specimens allow for a better understanding of the distribution patterns of all species.

**Figure 13.**
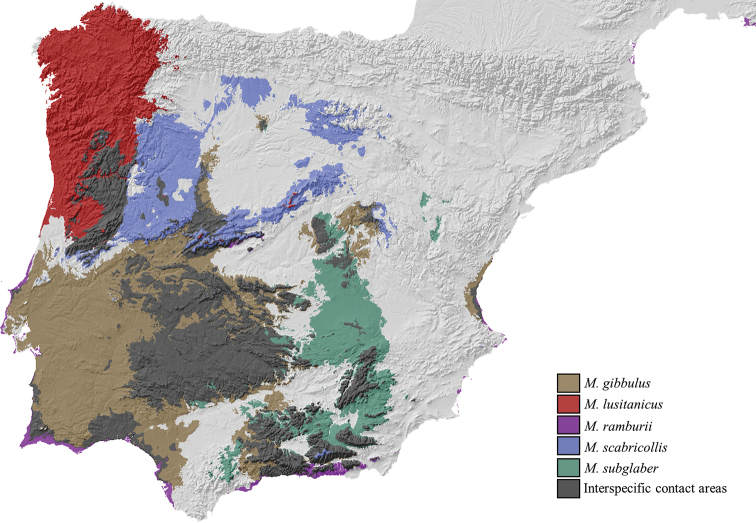
Map of the potential geographic distribution of the Iberian species of *Misolampus*, with the areas of high suitability (suitability > 0.7) depicted for all the species combined: *M. gibbulus* (orange), *M. lusitanicus* (red), *M. ramburii* (purple), *M. scabricollis* (dark blue), *M. subglaber* (green). Dark grey areas correspond to interspecific contact areas. The areas of low suitability for the occurrence of Iberian species of *Misolampus* are represented in pale grey.

Our data show some level of sympatry among several species pairs (i.e., *M. gibbulus* – *M. scabricollis*, *M. ramburii* – *M. subglaber*), even with cases of microsympatric distribution. These levels of sympatry among ecologically similar, phylogenetically closely related taxa are not common because of demographic processes such as competitive exclusion (Hardin 1960; Waters et al. 2013). Assuming the existence of ecological niche overlap among species pairs of *Misolampus*, areas of sympatric distribution can be explained by simultaneous colonisation from their respective glacial refugia, rapidly spreading into areas with favourable habitats while population densities are still very low, allowing for the establishment of two species (Recuero and García-París 2011; Escoriza et al. 2016; Yackulic 2017). In this way, areas traditionally considered glacial refugia in the Iberian Peninsula (e.g., southern Portugal, Atlantic Coasts of Galicia and Northern Portugal, southeastern Spain) (Martínez-Solano et al. 2006; Sánchez-Montes et al. 2018), where population sizes would have remained high, and thus favouring processes as competitive exclusion, are typically inhabited by a single species of *Misolampus*. The species distribution models show that the species of *Misolampus* present almost complementary potential distributions, supporting the hypothesis that current sympatry areas are the result of recent contact among taxa. The map including highest suitability areas (suitability > 0.7) for all the Iberian species combined (Fig. 13), shows that most of the high suitable areas do not overlap. Species suitable areas remain mainly restricted to the following regions: *M. gibbulus* in the southwest, *M. lusitanicus* in the northwest, *M. ramburii* in the southern coasts, *M. scabricollis* over the northern Iberian Plateau, and *M. subglaber* in the southeastern areas of the Iberian Plateau and along the Betic Mountains.

Additionally, our results indicate the presence of the genus in geographical areas where it had never been recorded. The absence of *Misolampus* from most part of the Sistema Ibérico mountain chain is particularly striking, considering the huge extension of favourable forest habitats. The recent finding of *M. subglaber* in the province of Cuenca, as well as the published record from the province of Valencia (Ibañez Orrico 2002), suggests that further populations could be discovered with more intensive sampling, at least in the southern parts of the Sistema Ibérico. Similarly, our records of *M. scabricollis* from the provinces of Burgos and Guadalajara are relatively close to the Sistema Ibérico mountains, where the species could be present, but still undetected. Similar cases of long undetected presence of arthropod species in the Sistema Ibérico have been recently published (Valladares et al. 2000; Pérez-Onteniente et al. 2015; Ruiz 2015; Recuero and Rodríguez-Flores 2019).

**Figure 14.**
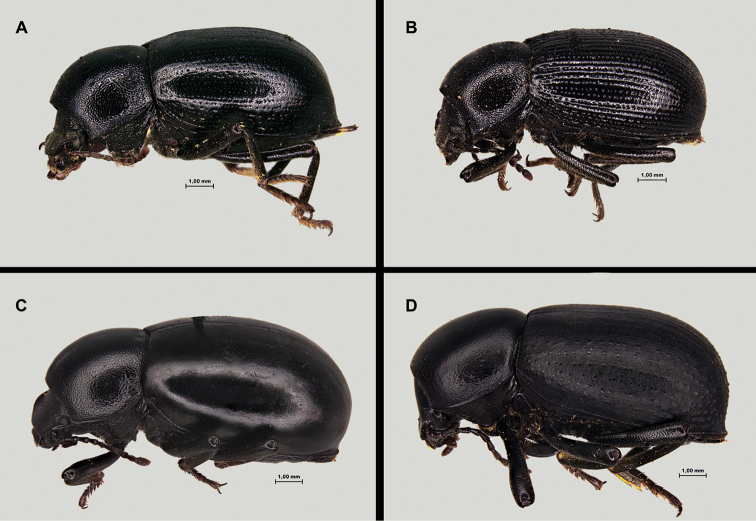
Lateral view of specimens of *Misolampus***A***Misolampus ramburii* from Málaga **B***Misolampus gibbulus* from Santa Elena (Jaén) (MNCN_Ent 270070) **C***Misolampus subglaber* from Cazorla (Jaén) (MNCN_Ent 270037) **D***Misolampus goudotii* from Menorca Island (MNCN_Ent 270032). Note the marked differences in sculpture of elytra among all four species.

Field data collection, although essential, has the disadvantage of being limited across space, time and taxa, which can constitute a constraint for biodiversity monitoring and conservation (Kuussaari et al. 2009; Meineke et al. 2019). Lack of information on changes in biodiversity through time and on the direction of these changes can make it difficult to identify and counteract negative impacts derived from disturbances (Magurran et al. 2010). However, scientific collections hold in a single location an enormous amount of information regarding a wide variety of taxa and, even though their potential has been historically under-appreciated, they are currently considered invaluable resources for biological studies (Meineke et al. 2019; Salvador and Cunha 2020). These collections provide data on taxon distributions over a vast time, offering a unique perspective on species response to habitat loss and fragmentation, land use intensification or climate change, thus providing critical information to reconstruct species decline and develop conservation strategies (Ponder et al. 2001; Suarez and Tsutsui 2004; Grixti et al. 2009; Doadrio et al. 2019).

**Figure 15.**
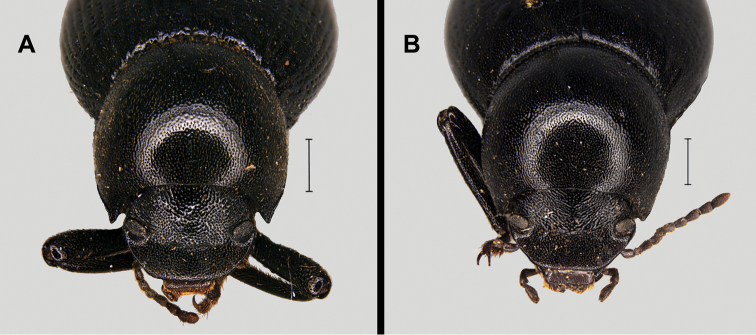
Fronto-dorsal view of specimens of *Misolampus***A***Misolampus gibbulus* from Santa Elena (Jaén) (MNCN_Ent 270070) **B***Misolampus ramburii* from Málaga (MNCN_Ent 270037). Note the differences between the two species in the shape of the anterior angles of the prothorax. Scale bars: 1 mm.

The way scientific collections were gathered and the form in which they have been preserved, offer a vast array of possibilities for past-present comparisons in this era of biodiversity loss (Short et al. 2018). Large entomological collections are often formed by the addition of multiple smaller collections (Cambefort 2006; Doadrio et al. 2019). Each taxonomist’s collection is a summary of the general biodiversity knowledge at the time, for each of their groups of study. In this sense, scientific collections represent temporal windows opened to a now unreachable past biodiversity, and access to them should be essential and promoted (Mantle et al. 2012; Short et al. 2018).

**Figure 16.**
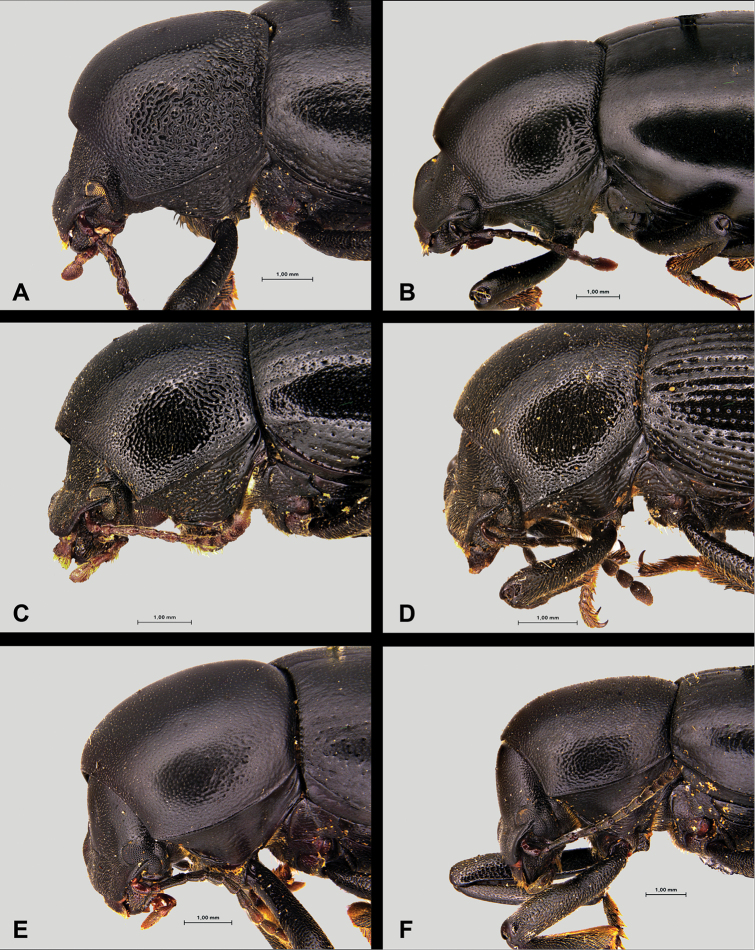
Lateral view of the head and prothorax of specimens of *Misolampus***A***Misolampus scabricollis* from Puerto de Navacerrada (Madrid) (MNCN_Ent 270049) **B***Misolampus subglaber* from Cazorla (Jaén) (MNCN_Ent 270209) **C***Misolampus ramburii* from Málaga (MNCN_Ent 270037) **D***Misolampus gibbulus* from Santa Elena (Jaén) (MNCN_Ent 270070) **E***Misolampus goudotii* from Menorca Island (MNCN_Ent 270032) **F***Misolampus goudotii* from Iguermalen, Beni Mesdui (Rif Mountains, Morocco) (MNCN_Ent 270188). Note the marked differences in sculpture and anterior angles of pronotum among all five species represented. Photographs **E, F** represent some of the geographic variability observed within *M. goudotii*.

The saproxylic nature of *Misolampus* calls into question their conservation status, since saproxylic beetles have been identified as a highly threatened animal assemblage due to habitat loss derived from logging and the decline of veteran trees throughout the landscape (Davies et al. 2008; Ricarte et al. 2009; Nieto and Alexandre 2010; Marcos García and Galante 2013; García-López et al. 2016; García et al. 2018). Despite the potential threats to which the species of *Misolampus* can be subjected to, their current level of threat has not been evaluated within the frame of the regional IUCN Red List of Mediterranean saproxylic beetles (García et al. 2018).

However, our comparison of historical data with recent records to assess the current population trends of the species of *Misolampus*, reveals that their distribution ranges show no reduction in the last century, since these species currently persist in most areas of historical occurrence. This fact, combined with the addition of new recent records for some of the species, enables us to state that, from a general perspective, the species of *Misolampus* are not in decline, but rather seem to exhibit an adequate conservation status. This status could be further guaranteed, because the distribution range of all species of *Misolampus* include numerous protected areas (National and Natural Parks, Natura 2000 protected areas; see https://www.miteco.gob.es/es/biodiversidad/servicios/banco-datos-naturaleza/informacion-disponible/ENP.aspx), which could ensure to some extent the long-term persistence of these saproxylic beetles, if combined with the implementation of adequate agroforestry practices, consistent with the general strategies of saproxylic arthropods conservation from the Mediterranean forests ecosystems (Sánchez Martínez et al. 2012; Marcos García and Galante 2013; García et al. 2018).

Considering the habitat specificity of *Misolampus*, disjunct distribution records such as Ifni for *M. goudotii* (Fig. 4), or Cuenca and Valencia for *M. subglaber* (Fig. 12), can involve threats for the species conservation, derived from local population extinctions, which can be irrevocable in the case of isolated populations. However, disjunct distributions might be not only the result of a reduction of the species range (Teixeira et al. 2018), but also a consequence of recent expansion (Mas-Peinado et al. 2015). Distinguishing between these two situations is highly relevant when evaluating the conservation status of a given species (Hampe and Petit 2005).

**Figure 17.**
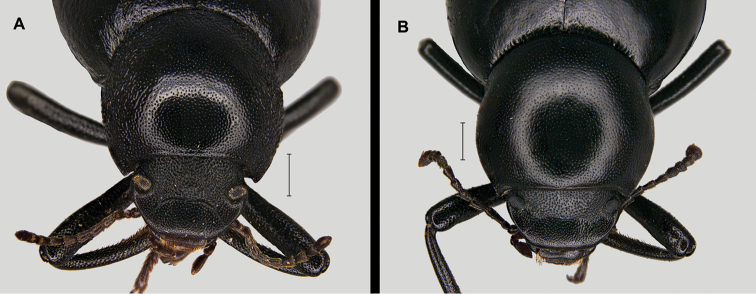
Fronto-dorsal view of specimens of *Misolampus***A***Misolampus scabricollis* from Puerto de Navacerrada (Madrid) (MNCN_Ent 270049) **B***Misolampus subglaber* from Cazorla (Jaén) (MNCN_Ent 270209). Note the differences in the lateral sculpture of pronotum. Scale bars: 1 mm.

## Supplementary Material

XML Treatment for Misolampus gibbulus

XML Treatment for Misolampus goudotii

XML Treatment for Misolampus lusitanicus

XML Treatment for Misolampus ramburii

XML Treatment for Misolampus scabricollis

XML Treatment for Misolampus subglaber
